# Investigation of the association between circulating inflammatory proteins and encephalitis risk in Europeans by two-sample Mendelian randomization analysis

**DOI:** 10.3389/fneur.2024.1450735

**Published:** 2025-02-11

**Authors:** Yanwei Liu, Xilong Wang, Qiang Zhao, Jun Wei, Shiqiang Yang

**Affiliations:** ^1^Department of Neurology, First People’s Hospital of Yibin, Yibin, China; ^2^Department of Neurology, Five People’s Hospital of Yibin, Yibin, Sichuan, China; ^3^Department of Neurosurgery, West China Hospital of Sichuan University, Chengdu, China

**Keywords:** circulating inflammatory proteins, viral encephalitis, acute disseminated encephalomyelitis, autoimmune encephalitis, Mendelian randomization

## Abstract

**Background:**

Cytokines are powerful immune response factors that operate at inflammation sites and are also found in the blood. Nevertheless, research on encephalitis and these circulating inflammatory proteins is quite limited.

**Methods:**

This study investigated the potential causal effects of 91 circulating inflammatory proteins on three different types of encephalitis using a two-sample Mendelian randomisation analysis. The data source for encephalitis was the latest Finngen_R12 dataset, released in 2024. The study investigated causal effects mainly using Steiger, MR-Egger, weighted median and inverse variance weighting (IVW) methods. In addition, sensitivity analyses were performed, including heterogeneity assessment, horizontal pleiotropy and leave-one-out techniques.

**Results:**

In this study, 91 circulating inflammatory proteins were subjected to MR analysis of causality with each of the three types of encephalitis. The results suggest that the inflammatory factors with a potential causal relationship with viral encephalitis are artemin, C-C motif chemokine 28, C-X-C motif chemokine 1, interleukin-10 and neurotrophin-3. Inflammatory factors potentially causally associated with acute disseminated encephalomyelitis are monocyte chemoattractant protein 2, interleukin-10 receptor subunit beta and matrix metalloproteinase-1. Inflammatory factors potentially causally associated with autoimmune encephalitis are C-C motif chemokine 28 levels and Macrophage inflammatory protein 1a levels.

**Conclusion:**

This study identifies potential causal effects of certain circulating inflammatory factors on susceptibility to three types of encephalitis. Although the exact mechanisms by which inflammatory proteins contribute to the pathogenesis of different encephalitis subtypes remain unclear, our findings provide new perspectives on these potential causal relationships.

## Introduction

Encephalitis has become a major global health burden due to high rates of disability and mortality. According to the Global Burden of Disease (GBD), encephalitis is one of the top ten causes of neurological Disability Adjusted Life Years (DALYs) worldwide. However, a specific aetiology can be identified in less than half of cases (1). A review of encephalitis infections published in 2016 highlighted viral infections as the main pathogen associated with encephalitis. In addition, acute disseminated encephalomyelitis and autoimmune encephalitis are common subtypes of encephalitis that are more commonly seen in clinical practice (2). Timely and definitive diagnosis of encephalitis in clinical practice is challenging because the non-specific symptoms of neuroencephalitis are similar to those of other neurological and psychiatric disorders. Oligoclonal banding (OCB) is a specific immunoglobulin G (IgG) banding pattern detected in cerebrospinal fluid (CSF) by isoelectric focusing electrophoresis (3). In autoimmune encephalitis, such as anti-NMDAR encephalitis, OCB status may be a potential prognostic biomarker. This means that the presence or absence of OCB may correlate with disease severity, response to treatment or long-term prognosis (5).

Cytokines are potent molecules of the immune response. They act at the site of inflammation and circulate in the bloodstream (6). Immune-mediated responses have been implicated in the pathogenesis of encephalitis. Recent studies have identified several cytokines that exhibit significant changes in plasma levels, including B-cell activating factor for B-cell proliferation, thymus and activation-regulated chemokines for T-cell chemoattraction, soluble CD40 ligand for Th2 cell-mediated responses, C5/C5a for complement activation, brain-derived neurotrophic factor for neuronal survival responses, and dipeptidyl peptidase 4, retinol-binding proteins, Dickkopf-associated proteins, and epidermal growth factor in response to environmental stimuli (7–9). The heightened concentrations of multiple cytokines observed in individuals with encephalitis underscore the significant role played by inflammatory and autoimmune processes (10). This inflammatory cascade is regulated by an intricate system of cells and signaling molecules, including cytokines and soluble receptors present in circulation (11). Consequently, identifying the genetic factors influencing the levels of inflammation-related circulating proteins could provide valuable knowledge regarding the pathophysiology and origins of various diseases. However, the clinical significance of these cytokines remains incompletely understood as a result of insufficient experimental and clinical research. Therefore, a thorough investigation into the potential causal association between circulating inflammatory proteins and encephalitis could elucidate the underlying biological mechanisms of encephalitis pathogenesis (12).

Mendelian randomization is a statistical technique that employs single nucleotide polymorphisms (SNPs) as instrumental variables (IVs) to establish causal relationships between exposure and outcome (13). Through random allocation during meiosis, SNPs are effectively categorized into groups based on genetic characteristics, resembling a randomized controlled trial. By reducing biases inherent in observational studies, MR strengthens the validity of causal inferences regarding exposure-outcome associations (14). However, current studies lack a comprehensive analysis of the potential causal relationship between circulating cytokine levels and different types of encephalitis. The data analysed in this study were derived from a recently published genome-wide association study (GWAS) of circulating inflammatory proteins. Data on encephalitis and subtypes as study endpoints were obtained from the most recent Finngen Consortium R12 study dataset, published in 2024. In this study, we investigated the potential causal association of 91 circulating inflammatory proteins with three types of encephalitis using two-sample MR analysis.

## Methods

### Study design

**Figure 1:**
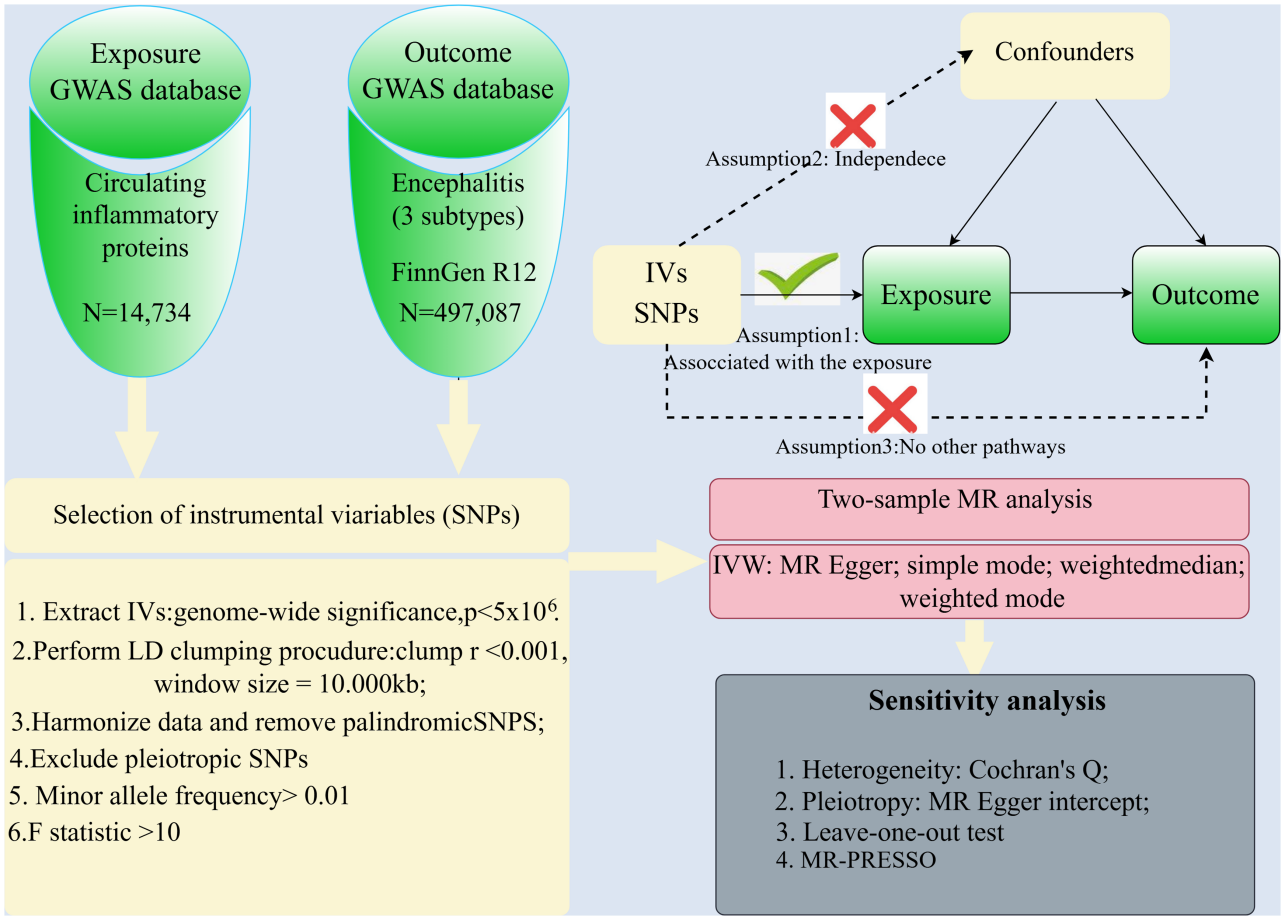
Overview of the study design and analysis.

This study is presented in accordance with the STROBE-MR guidelines (15). A schematic representation of the study design is depicted in Figure 1. This study was based on three research hypotheses: (1) Association hypothesis: the genetic variance (instrumental variable) must be strongly associated with the exposure factor under study. (2) Independence assumption: genetic variants are not associated with any potential confounders. (3) Exclusionary limiting assumption: genetic variation can only affect the outcome by influencing the exposure factor, and not by other means. This excludes the possibility that genetic variants may influence outcomes directly or through other risk factors. To fulfill these assumptions, the following steps were taken in this study: (1) Selection of instrumental variables: genetic variants significantly associated with exposure factors were selected through GWAS data. (2) Validating associations: Ensuring that the selected genetic variants were statistically significantly associated with the exposure factors. (3) Controlling for confounders: Sensitivity analyses using statistical methods such as MR-Egger regression, weighted median method and weighted mode method to detect and adjust for the effects of confounders. (4) Testing for pleiotropy: MR-Egger regression and other methods are used to test whether genetic variation may affect the results by other means.

The objective of this study was to conduct a thorough analysis of the potential genetic causal relationship between 91 circulating immune proteins and three types of encephalitis. To mitigate population stratification bias, participants in both the exposure and outcome groups were limited to individuals of European descent. Given the anonymized nature of the data and the non-invasive nature of the study, ethical approval was obtained from the Ethics Committee of the First People’s Hospital of Yibin. Nonetheless, this study adhered to the highest ethical standards for protecting participant privacy and data security when analyzing and reporting results.

### Source of outcome

Data on 91 circulating inflammatory proteins were collected from a recently published global genomic study conducted in 2023 involving 14,734 people of European ancestry. The study’s population was drawn from 11 different study cohorts, totaling multiple populations of European ancestry. The study utilized genome-wide protein quantitative trait locus (pQTL) analysis to investigate the genetic impact of inflammation-related proteins, specifically those measured using the Olink Target platform (16). Data on these 91 plasma inflammatory proteins, including pQTL findings, are available in the EBI GWAS catalog (registry numbers GCST90274758 to GCST90274848).

Summary statistics from the genome-wide association study (GWAS) of the 3 encephalitis species that served as study endpoints were obtained from the FinnGen consortium R12 release (Finngen_R12 THROMBANG). The FinnGen study was initiated in 2017 as a comprehensive national effort to combine genetic information from the Finnish BioBank with national registries’ Digital Health Records combined GWAS included a large cohort of 412,181 Finnish participants and analyzed 21,311,942 variants.

704 cases and 497,501 controls were identified for viral encephalitis according to the International Classification of Diseases ICD-9 (046.9) and ICD-10 (A86); 64 cases and 497,087 controls were identified for acute disseminated encephalomyelitis according to ICD-9 (323) and ICD-10 (G04.0); 966 cases and 497,087 controls were identified for autoimmune encephalitis according to ICD ICD-9 (323.6) and ICD-10 (G04.8).For additional information about the data, such as participant demographic characteristics, categories, and specific GWAS types, see Table 1.

**Table 1:** Source: Pooled data from genome-wide association studies of exposures and outcomes in this study.

**Exposure and outcome phenotypes**	**No. of cases**	**No. of controls**	**Sample** **size**	**Population**	**Year**	**PMID**	**Consortium**	**Build**
**Exposures**								
Circulating inflammatory proteins (91 subtypes)	14,734	NA	14,734	European	2023	37563310	NA	HG38/GRCh38
**Outcomes**								
Viral encephalitis	704	497,501	498,205	European	2024	NA	Finngen R12	HG38/GRCh38
Acute disseminated encephalomyelitis	64	497,087	497,151	European	2024	NA	Finngen R12	HG38/GRCh38
Autoimmune encephalitis	966	497,087	498,053	European	2024	NA	Finngen R12	HG38/GRCh38

### Instrumental variables selection

At the outset of this study, when selecting the instrumental variables, we were prepared to use 5 × 10^−8^ as a threshold for distinguishing significant differences in the exposure data. However, the results indicated the problem of insufficient instrumental variables for some exposure factors. Later, a more lenient threshold of 5 × 10^-6^ was used. The study assessed the directionality of the relationship between the remaining instrumental variables and the outcomes by applying a Steiger filter. Subsequently, disjunction disequilibrium was assessed utilizing a reference panel of 1,000 genomes with thresholds of *r*^2^ > 0.001 and clustering distances <10,000 kb (17). In order to ensure the reliability of the chosen SNPs, the F-statistic was computed to eliminate weak instrumental variables with *F*-values below 10.The F-statistic was calculated as F = R2(N-2)/(1-*R*^2^), where *R*^2^ represents the proportion of variation explained by the genetic instrument, and N is the effective sample size of the SNP-exposure-associated GWAS study. The R2 value was calculated as 2 × MAF (1-MAF) beta2, where beta represents the estimate of the effect of exposure genetic variation in standard deviation (SD), and MAF represents the minor allele frequency (18). Subsequently, SNPs that may be associated with risk factors related to structural changes in the brain were excluded using PhenoScanner. This exclusion included neurological and psychiatric disorders, hypoxaemia and other possible confounding variables. The resulting SNPs were then used in a Mendelian randomization analysis (19).

The analyses were performed using the TwoSample MR (version 0.5.7) package in R (version 4.3.2) and Free Statistics software version 2.0 (Beijing, China). Effect sizes (*β*) and 95% confidence intervals (CI) were used to express MR estimates.

### Statistical analysis

In our analysis, we assessed the contribution of each cytokine to the variance explained by the main instrumental variable and eliminated weak instrumental variables based on the F statistic. Specifically, effect size and standard error estimates were obtained for 91 circulating cytokines and 3 encephalitis species, respectively. Individual Mendelian randomization estimates were subsequently derived using the Wald ratio and Delta methods. To evaluate the relationship between genetically determined circulating cytokine levels and encephalitis, we aggregated MR estimates for individual SNPs using IVW techniques. IVW is the most commonly used method in Mendelian randomization analyses, which combines the effect sizes (Wald ratios) of multiple genetic variants in a fixed effects meta-analysis, where each ratio is weighted as the inverse of the variance of the association between the SNP and the outcome. In MR analyses, each SNP was treated as a separate “study” and was meta-analyzed under the fixed effects model. The weighted median method is considered robust under the assumption that a substantial portion of instrumental variables are valid, particularly when the proportion of instrumental variables demonstrating horizontal pleiotropy is less than 50 percent. Conversely, the MR-Egger method is deemed reliable when more than half of the instrumental variables display horizontal pleiotropy. To evaluate horizontal pleiotropy, sensitivity Mendelian randomization analyses were performed under various assumptions utilizing the weighted median, MR-Egger, and MR-PRESSO methods.

In order to enhance the reliability of our results, we performed sensitivity analyses utilizing various methods such as the sample median method, weighted median method, MR-Egger regression, and maximum likelihood method. Initial assessment of heterogeneity was conducted through Cochran’s Q analysis, with a significance level of *p* > 0.05 indicating the presence of heterogeneity. In cases of heterogeneity, the fixed-effects IVW method was employed. The intercept of MR-Egger regression serves as a valuable tool for identifying horizontal pleiotropy. The extended IVW method was employed as the primary analytical technique in this study, with the MR-Egger intercept utilized to evaluate potential horizontal pleiotropy. Additional sensitivity analyses were conducted to mitigate potential confounders. Following the exclusion of confounding SNPs, further MR analyses were carried out on the significant findings from the initial analyses to validate the preliminary results. All MR analyses were conducted using the TwoSample MR, Mendelian Randomization, and MR-PRESSO packages in R (version 4.3.2).

## Results

This study utilized genetic variants as instrumental variables to assess potential causal associations between circulating inflammatory proteins and three types of encephalitis. We selected genetic variants with strong associations with exposure and outcome that showed consistent patterns of association in two independent samples. This consistency increased our confidence in the validity of the genetic instrumental variables and reduced potential bias due to confounders or reverse causation. In this study, our exposure and outcome samples came from studies with different national cohorts and there were no overlapping individuals between them. This was done to maintain the independence of the two samples, thus avoiding bias in MR estimation due to sample overlap. By using two independent samples, we were able to more accurately estimate the potential causal effect of genetic variation on the relationship between circulating inflammatory proteins and encephalitis subtypes.

### Genetic tools for 91 circulating inflammatory proteins used for exposure

Figure 1 shows the complex design and flowchart of the study. Under certain conditions, we extracted SNPs with significant differences in 91 inflammatory proteins as instrumental variables. Those SNPs with weak instrumental qualities, as indicated by F-statistic values below 10, were excluded. PhenoScanner was then used to eliminate potentially confounding SNPs, resulting in a final selection of 6-21 SNPs with F-statistic values ranging from 22.8 to 99.6, as shown in Table 2.

**Table 2:** Mendelian randomization analysis between circulating inflammatory proteins and different types of encephalitis.

**Outcomes**	**NSNPs**	Inverse variance weighted*			**Weighted median**			**MR-Egger**		
**Exposures**		**Beta**	**SE**	***P***	Beta	SE	*P*	Beta	SE	*P*
**Viral encephalitis**										
Artemin	17	**0.431**	**0.201**	**0.031**	0.623	0.276	0.023	0.891	0.513	0.103
C-C motif chemokine 28	20	**-0.471**	**0.213**	**0.027**	-0.169	0.315	0.591	-0.387	0.457	0.409
C-X-C motif chemokine 1	8	**0.423**	**0.177**	**0.017**	0.395	0.190	0.037	0.254	0.269	0.383
Interleukin-10	18	**0.470**	**0.166**	**0.004**	0.353	0.236	0.134	0.639	0.343	0.081
Neurotrophin-3	16	**0.293**	**0.131**	**0.005**	0.383	0.319	0.231	0.441	0.524	0.414
**Acute disseminated encephalomyelitis(ADEM)**										
Monocyte chemoattractant protein 2	24	**0.370**	**0.246**	**0.013**	0.228	0.268	0.395	0.249	0.301	0.416
Interleukin-10 receptor subunit beta	19	**-0.576**	**0.362**	**0.011**	-0.379	0.408	0.352	-0.551	0.547	0.328
Matrix metalloproteinase-1	26	**0.825**	**0.528**	**0.015**	2.112	0.749	0.004	1.780	0.935	0.077
**Autoimmune encephalitis**										
C-C motif chemokine 28	20	**-0.573**	**0.182**	**0.002**	-0.755	0.239	0.001	-0.468	0.387	0.241
Macrophage inflammatory protein 1a	13	**0.282**	**0.133**	**0.034**	0.148	0.144	0.304	0.134	0.214	0.544

Abbreviations: NSNPs Number of single-nucleotide polymorphisms, SE standard error, A *p* value < 0.05 was considered nominally significant.

*IVW; Inverse variance weighted，The IVW method is the most dominant method in Mendelian randomization analysis and its *P* < 0.05 indicates a statistically significant difference.

**Figure 2:**
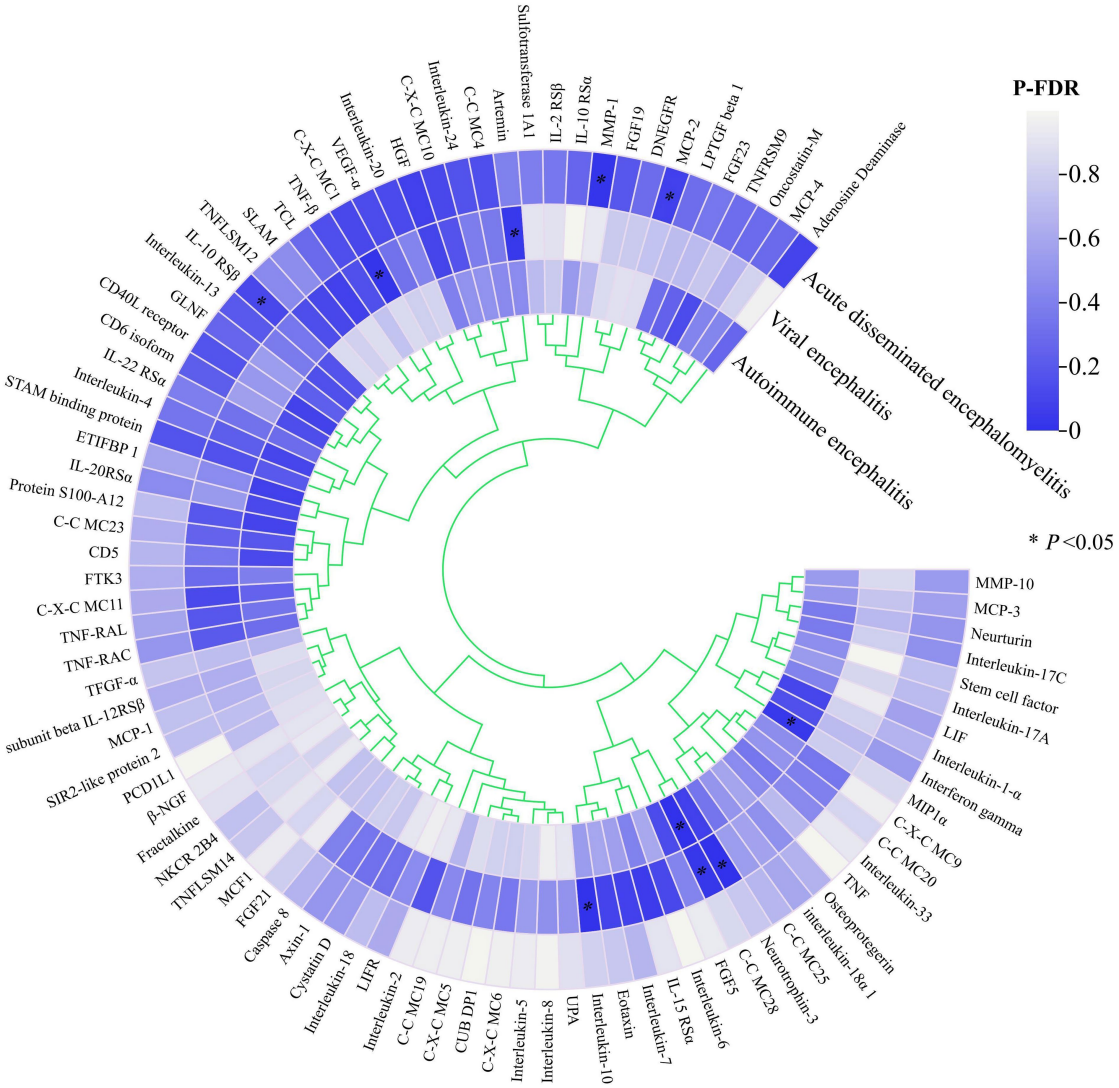
Heat map of all exposures and endings.

Figure 2 illustrates the potential causal relationship between the circulating inflammatory factor profiles predicted by all 91 genes and three different types of encephalitis. All P values corrected for FDR were clustered and colour coded according to the direction of effect.

### Estimates of the causal relationship between circulating inflammatory proteins and viral encephalitis

**Figure 3:**
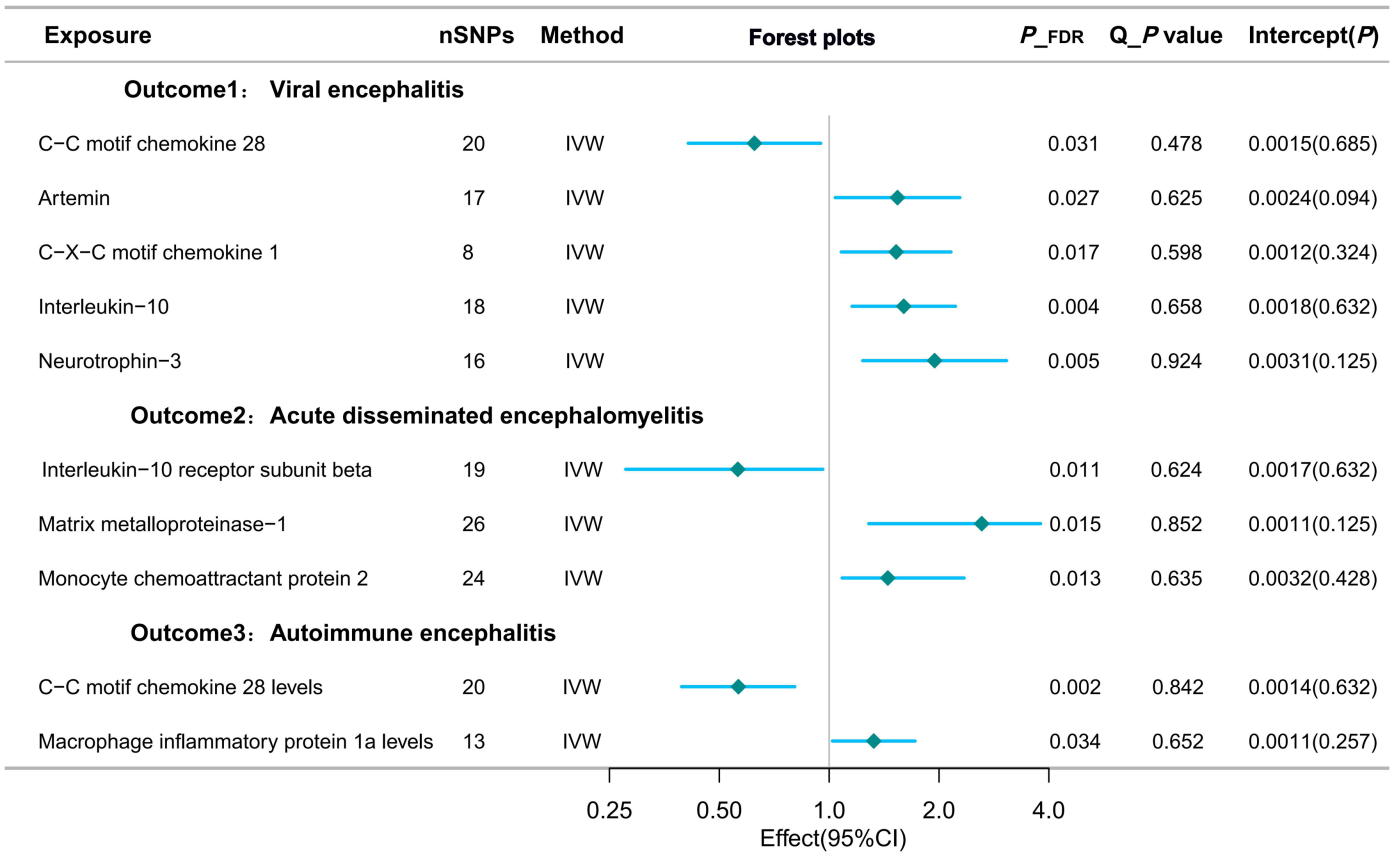
Forest plots of potential causal exposures and outcomes.

In the most recent GWAS dataset of circulating inflammatory proteins, published in 2023, our team identified 91 isoforms as exposures of interest. We then performed Mendelian randomisation analysis on the viral encephalitis dataset from the latest 2024 release of the Finnegan Consortium R12 version. Of these, six potential causal associations showed statistically significant differences in the Mendelian analyses after adjustment for false discovery rate (FDR).Specifically, genetically predicted C-C motif chemokine 28 levels (OR=0.624; 95% CI=0.411-0.948, *p* = 0.027) were potentially negatively associated with viral encephalitis in the IVW approach. However, artemin levels (OR=1.539; 95% CI=1.039-2.281, *p* = 0.038), C-X-C motif chemokine 1 levels (OR=1.526; 95% CI=1.079-2.157, *p* = 0.017), interleukin-10 levels (OR=1.601; 95% CI=1. 155-2.219, *p* = 0.005) and neurotrophin-3 levels (OR=1.945; 95% CI=1.236-3.061, *p* = 0.004) were positively associated with the development of viral encephalitis (Table 2; Figure 3).

### Estimates of the causal relationship between circulating inflammatory proteins and acute disseminated encephalomyelitis

When the study outcome was replaced with acute disseminated encephalomyelitis for the analyses, we identified three potential causalities that showed statistically significant differences after adjusting for FDR. When targeting acute disseminated encephalomyelitis as an outcome subtype, three potential causal associations showed statistically significant differences after adjusting for FDR. In particular, the IVW approach suggests that genetically predicted monocyte chemoattractant protein 2 (OR=1.448; 95% CI=1.085-2.343, *p* = 0.013) and matrix metalloproteinase-1 showed a potential positive association (OR=3.619; 95% CI=1.284-10.205, *p* = 0.015). However, interleukin-10 receptor subunit beta levels (OR=0.562; 95% CI=0.277-0.961, *p* = 0.011) were potentially negatively associated with acute disseminated encephalomyelitis (Table 2; Figure 3).

### Estimates of the causal relationship between circulating inflammatory proteins and autoimmune encephalitis

Using autoimmune encephalitis as the outcome for MR analysis, this study identified 2 potential causal relationships. Gene-predicted C-C motif chemokine 28 levels (OR=0.564; 95% CI=0.394-0.805, *p* = 0.002) were potentially negatively associated with autoimmune encephalitis in the IVW analysis method. However, levels of macrophage inflammatory protein 1a (OR=1.325; 95% CI=1.021-1.719, *p* = 0.034) showed a potential positive association with autoimmune encephalitis (Table 2; Figure 3).

### Sensitivity analysis

SNPs failing to meet the threshold of genome-wide significance (*p* < 5 × 10–8) for the outcome variable were initially omitted from the analysis. The directionality of the associations between the remaining instrumental variables and the outcome was then evaluated using Steiger filtering. Correlations with raw *p* values below 0.05, though not meeting the aforementioned thresholds, were deemed statistically significant. In Mendelian randomization analyses, any instrumental variables flagged as spurious by Steiger filtering (indicating that the SNP explained a greater proportion of the variance in the outcome compared with exposure) were excluded. In order to evaluate the existence of horizontal pleiotropy, sensitivity Mendelian randomization analyses were performed utilizing multiple methodologies including weighted median, MR-Egger, and MR-PRESSO. The findings from these analyses were concordant with those obtained from the inverse variance weighted analyses. The weighted median method operates under the assumption that a substantial portion of the instrumental variables are reliable, and demonstrates resilience when the proportion of potentially biased instrumental variables is below 50 percent. It should be emphasized that the presence of heterogeneity was initially assessed by Cochran Q analysis. The *p*-values of the Cochran Q statistics were all greater than 0.05, indicating the absence of heterogeneity. This study also used the MR-PRESSO global test to assess the presence of horizontal pleiotropy among all instrumental variables. The results showed that the exposure-outcome analysis tests with potential causality all suggested a *p*- value greater than 0.05, suggesting no horizontal pleiotropy (Table 3).

**Table 3:** Heterogeneity and pleiotropy tests of the causal effects of Circulating inflammatory proteins on different types of encephalitis.

**Outcomes**	**NSNPs**	**Cochrane’s Q test**		**MR-Egger intercept test**		**MR-PRESSO**		**Steiger test**	**F-statistics**
**Exposures**		**Q-value**	***P***	**Intercept**	***P***	**Globle test *P***	**Cor-*P***		
**Viral encephalitis**									
Artemin	17	91.2	0.478	0.0015	0.685	0.621	-	True	16.5-99.7
C-C motif chemokine 28	20	82.3	0.625	0.0024	0.094	0.203	-	True	20.1-79.7
C-X-C motif chemokine 1	8	84.1	0.598	0.0012	0.324	0.181	-	True	19.2-112.3
Interleukin-10	18	102.6	0.658	0.0018	0.632	0.235	-	True	16.4-845.1
Neurotrophin-3	16	70.9	0.924	0.0031	0.125	0.632	-	True	18.8-95.7
**Acute disseminated encephalomyelitis**									
Monocyte chemoattractant protein 2	19	68.8	0.624	0.0017	0.632	0.157	-	True	19.5-210.1
Interleukin-10 receptor subunit beta	26	76.5	0.852	0.0011	0.125	0.369	-	True	18.2-85.7
Matrix metalloproteinase-1	24	82.3	0.635	0.0032	0.428	0.236	-	True	16.8-116.7
**Autoimmune encephalitis**									
C-C motif chemokine 28	20	66.5	0.842	0.0014	0.632	0.635	-	True	18.1-89.6
Macrophage inflammatory protein 1a	13	79.3	0.652	0.0011	0.257	0.576	-	True	17.6-102.3

Abbreviations: NSNPs Number of single-nucleotide polymorphisms, Q-test p-value > 0.05 suggests no heterogeneity. MR-Egger, intercept test P-value > 0.05 suggests no horizontal pleiotropy. Cor-*P,* Outlier-Corrected P.

The intercept of the MR-Egger regression was utilized in this investigation to evaluate the potential existence of horizontal pleiotropy. All MR-Egger intercept test statistics pertaining to causality yielded p-values exceeding 0.05, suggesting the lack of horizontal pleiotropy. To validate the identified causality, a reverse MR analysis was conducted on the aforementioned statistically significant findings. No evidence of encephalitis impacting cytokine levels was observed (Supplementary Table S1). Scatter plots, forest plot, funnel plots, and leave-one-out sensitivity analysis plots for all exposure-outcome relationships with potential causality are shown in Supplementary Figures S1–S40.

## Discussion

The present study used a recently published large-scale GWAS dataset to perform a comprehensive two-sample Mendelian randomisation analysis to elucidate potential causal relationships between circulating inflammatory proteins and three different forms of encephalitis at the genetic level. Our results indicate that artemin, C-C motif chemokine 28, C-X-C motif chemokine 1, interleukin-10 and neurotrophin-3 are potentially causal inflammatory factors. Inflammatory factors potentially causally associated with acute disseminated encephalomyelitis are monocyte chemoattractant protein 2, interleukin-10 receptor subunit beta and matrix metalloproteinase-1. Inflammatory factors that may be causally associated with autoimmune encephalitis are C-C motif chemokine 25 levels and hepatocyte growth factor levels.

Viral encephalitis is the predominant subtype of encephalitis in numerous geographical areas. Cytokines, encompassing both proinflammatory and anti-inflammatory types, serve as pivotal regulators in the host’s reaction to viral infection, pathogenesis, and disease progression. The host’s cytokine responses are genetically determined and reflect the intricate array of inter-individual variations in immune responses to viral infections (3, 20). Currently, supportive symptomatic therapy remains the primary treatment option for patients with viral encephalitis, as effective targeted therapies have not been developed. The morbidity and mortality rate among patients with viral encephalitis is approximately 30%, with over half of survivors experiencing various neuropsychiatric sequelae (21). Neuroleptic viral infections causing viral encephalitis have garnered increased attention due to their significant mortality and disability rates (2). However, existing studies have not yet comprehensively analyzed inflammatory proteins. Limited observational studies conducted over the past decades have shown a correlation between certain inflammatory proteins and the risk of viral encephalitis (22). Through this study, it was confirmed that Caspase 8 levels, Interleukin-10 levels, Interleukin-7 levels, and TNF-beta have a positive correlation on the occurrence of viral encephalitis at the genetic level. However, the findings of these studies are often inconsistent due to selection biases in case sample selection and subsequent analyses inherent in observational studies (23, 24). Hence, the practical application of these biomarkers as reliable diagnostic and prognostic indicators is not feasible (25). This research employed a two-sample Mendelian randomization methodology to establish strong causal relationships, mitigating potential confounding factors and eliminating the possibility of reverse causation. Furthermore, we utilized data from comprehensive global genomic studies accessible to the public, encompassing a substantial cohort of individuals with viral encephalitis and control subjects. The analysis of inflammatory proteins was conducted using the most up-to-date and extensive genomic datasets available. This provides strong and solid evidence for exploring potential causal relationships.

Furthermore, as a secondary objective of this research, we conducted a thorough Mendelian randomization analysis on acute disseminated encephalomyelitis, autoimmune encephalitis, and inflammatory proteins. Acute disseminated encephalomyelitis (ADEM) is a condition characterized by immune-mediated inflammation of the central nervous system, leading to widespread demyelination primarily in the white matter of the brain and spinal cord (26). This inflammatory response is typically initiated by viral infections or vaccinations. ADEM is typically precipitated by viral infection or vaccination, presenting with acute encephalopathy characterized by multifocal neurological manifestations and deficits. While the condition can manifest at any age, it is more prevalent in pediatric populations (27). Due to the lack of definitive biomarkers, the diagnosis of ADEM relies on clinical and imaging criteria. Treatment options for ADEM are not standardized, with most approaches involving non-specific immunosuppressive therapy (28). As of present, there is a lack of randomized controlled trials investigating the treatment of ADEM in both pediatric and adult populations. The etiology of ADEM remains unclear, with current research indicating that microbial infections may trigger an autoimmune response targeting myelin through molecular mimicry (29). Alternatively, ADEM may result from the activation of pre-existing myelin-reactive T-cells via a non-specific inflammatory mechanism (30). Our study aims to conduct a thorough analysis of the relationship between ADEM and inflammatory proteins using Mendelian randomization analysis. The results suggest that Beta-nerve growth factor levels, Cystatin D levels, Interleukin-7 levels, Latency-associated peptide transforming growth, factor beta 1 levels, Neurotrophin-3 levels and ADEM have a potential causal relationship. These findings will provide some new directions and ideas for further mechanistic studies, clinical diagnosis and treatment.

The relationship between IL-7 and LPTF-B1 and encephalitis involves complex immunoregulatory mechanisms. Biologically, this phenomenon is of interest because different types of encephalitis may involve different immune pathways. In ADEM and viral encephalitis, cytokines may play different roles. For example, certain cytokines may promote an inflammatory response in viral encephalitis and increase the chance of morbidity, whereas in ADEM they may help to reduce inflammation and decrease the chance of morbidity (31). This depends on how well they activate or inhibit specific immune pathways. Current studies have found that common pro-inflammatory factors are IL-1*β*, IL-2, IL-6, IL-12, IL-17, IL-18, TNF-*α*, IFN-*γ*, TNF-β andCCL2, etc. And the common anti-inflammatory factors are IL-4, IL-10, IL-11, IL-13, TGF-β, IL-25, IL-37, IL-35, PGE2 and sTNFR, etc. These cytokines play complex roles in different immune responses and inflammatory processes, and they can regulate each other and work together to maintain the balance of the immune system (32). In certain disease states, the balance between pro-inflammatory and anti-inflammatory factors may be disrupted, leading to excessive or inadequate inflammatory responses. Understanding the mechanism of action of these cytokines is essential for the development of drugs for the treatment of inflammatory diseases. These findings offer valuable insights for further research on the mechanisms, diagnosis, and treatment of autoimmune encephalitis. As the screening and functional characterization of susceptibility genes progresses, the significance of these genetic variants in encephalitis episodes, as well as their interactions with other genes and the environment, will become more evident (33, 34). These advancements are anticipated to offer valuable insights into the immunological mechanisms involved in the development of encephalitis, and to facilitate more accurate diagnosis and treatment based on host genotypes. Functional genomics has opened up exciting new frontiers in elucidating the genetic architecture of encephalitis immunity and is now ripe for the picking.

### Advantages and limitations

This study’s primary strength resides in its utilization of a two-sample Mendelian randomization methodology to investigate potential causal associations between 91 circulating inflammatory factors and three distinct forms of encephalitis. Noteworthy strengths encompass the substantial sample size, the employment of various Mendelian randomization techniques to guarantee the reliability of results, and the utilization of GWAS data to mitigate confounding variables. The circulating inflammatory proteins utilized as exposures in this study were sourced from the GWAS dataset of the most recent meta-analysis released in 2023, representing the most comprehensive circulating protein data currently accessible. Furthermore, the study used multiple test correction methods: (1) e.g. Bonferroni correction, which adjusts the *p*-value threshold to control for false discovery rate (FDR). (2) Choose robust statistical methods: e.g., weighted median method or MR-Egger regression, which are resistant to outliers and genetic instrumental variables that violate the independence assumption. (3) Perform sensitivity analyses: Verify consistency of results by leave-one-out analysis or by repeating the analysis using different sets of genetic variants. (4) Transparent reporting of all analyses performed, including those results that were not statistically significant, will help to assess the credibility of the results.

This study used multiple methods, including weighted median, MR-Egger, and MR-PRESSO, to assess horizontal pleiotropy and perform sensitivity analyses. These methods were consistent with the results of the inverse variance weighted analyses, which reinforces our confidence in the results. Although the point estimates were similar in nature, the differences in standard errors primarily reflected the sensitivity of each method to outliers and potential horizontal pleiotropy. The differences observed in this study are primarily due to differences in how these methods handle independence and outliers in genetic instrumental variables. For example, the MR-Egger and MR-PRESSO methods accounted for possible horizontal pleiotropy when calculating SE, which may have led to larger SEs. In addition, MR-PRESSO adjusted the analysis by excluding outliers, which may explain the further increase in SE compared to the other methods. Despite these differences, the consistency of all methods demonstrates the robustness of the results of this study. Our results are not dependent on a single analytical method, but have been validated by multiple methods. We hope that these explanations help the reader to understand the impact of different methods on the significance findings and enhance the credibility of our findings.

However, it is important to acknowledge several limitations of this study. Firstly, Mendelian randomization evaluates the long-term impacts of exposure, while the levels of inflammatory proteins were measured solely at the time of enrollment, failing to consider temporal dynamics. Second, this research predominantly focused on epidemics of European descent identified in existing Genome-Wide Association Studies (GWAS), thereby restricting the applicability of the findings to other populations. Third, the study was unable to delve deeper into the distinct impacts of various subtypes of outcomes. This limitation arises from the potential development of different forms of encephalitis based on factors such as the origin of the infection and host genetics. Fourth, our analyses concentrated solely on levels of circulating inflammatory proteins, neglecting the potential for the local tissue cytokine environment to provide a more accurate representation of the inflammatory response in cases of encephalitis. Our inability to elucidate the potential dynamic changes in inflammatory protein levels during the clinical progression of encephalitis highlights a limitation in our study. Conducting further research to address these limitations will strengthen the validity of genetic studies on the inflammatory protein milieu in various subtypes of encephalitis. Consequently, additional experiments are necessary to clarify the underlying biological mechanisms elucidated in this study.

## Conclusion

In summary, our research employed Mendelian randomization to examine recently published Genome-Wide Association Study data from a substantial cohort, elucidating potential causal connections between 91 circulating inflammatory proteins and three distinct subtypes of encephalitis. Notably disparate causal relationships were observed for various inflammatory proteins across the three encephalitis subtypes. While the precise mechanisms by which inflammatory proteins contribute to the pathogenesis of encephalitis remain unclear, our results offer novel perspectives on the potential causal link between these entities. This will provide new directions for further in-depth studies.

## Data Availability

Publicly available datasets were analyzed in this study. The raw data required in this study can be extracted from the GWAS online website. The specific address is shown in Table 1. For further inquiries, please contact the first author or corresponding author.
