# Supplementary material for: Investigation of the association between circulating inflammatory proteins and encephalitis risk in Europeans by two-sample Mendelian randomization analysis
Source: Front Neurol. 2025 Feb 11;15:1450735. doi: 10.3389/fneur.2024.1450735 (PMC11850273; doi:10.3389/fneur.2024.1450735)
Supplement: Supplementary file 1 [file Table_1.docx]

**Supplementary Table 1** Inverse Mendelian randomization of circulating inflammatory proteins with three encephalitis subtypes.

| **Exposures** | **NSNPs** | **Inverse variance weighted** | | | **Weighted median** | | | **MR‐Egger** | | |
| --- | --- | --- | --- | --- | --- | --- | --- | --- | --- | --- |
| **Outcomes** |  | Beta | SE | *P* | Beta | SE | *P* | Beta | SE | *P* |
| **Viral encephalitis** |  | | | | | | | | | |
| Artemin | 4 | 0.012 | 0.021 | 0.528 | 0.012 | 0.024 | 0.594 | 0.012 | 0.071 | 0.874 |
| C-C motif chemokine 28 | 6 | -0.004 | 0.018 | 0.814 | 0.006 | 0.022 | 0.775 | 0.015 | 0.039 | 0.722 |
| C-X-C motif chemokine 1 | 8 | 0.011 | 0.017 | 0.526 | 0.011 | 0.021 | 0.598 | 0.015 | 0.041 | 0.729 |
| Interleukin-10 | 6 | -0.003 | 0.023 | 0.896 | -0.005 | 0.028 | 0.845 | -0.049 | 0.093 | 0.624 |
| Neurotrophin-3 | 32 | -0.004 | 0.019 | 0.842 | 0.005 | 0.023 | 0.846 | 0.023 | 0.046 | 0.652 |
| **Acute disseminated encephalomyelitis（ADEM）** |  | | | | | | | | | |
| Monocyte chemoattractant protein 2 | 5 | -0.001 | 0.022 | 0.953 | -0.002 | 0.015 | 0.902 | -0.004 | 0.031 | 0.912 |
| Interleukin-10 receptor subunit beta | 4 | -0.002 | 0.013 | 0.871 | -0.008 | 0.020 | 0.693 | -0.001 | 0.042 | 0.798 |
| Matrix metalloproteinase-1 | 4 | 0.009 | 0.013 | 0.475 | 0.014 | 0.345 | 0.743 | -0.012 | 0.058 | 0.746 |
| **Autoimmune encephalitis** |  | | | | | | | | | |
| C-C motif chemokine 28 | 7 | 0.002 | 0.019 | 0.873 | -0.004 | 0.024 | 0.922 | -0.006 | 0.038 | 0.882 |
| Macrophage inflammatory protein 1a | 6 | 0.009 | 0.024 | 0.698 | 0.003 | 0.029 | 0.917 | 0.054 | 0.072 | 0.493 |

Abbreviations:NSNPs Number of single-nucleotide polymorphisms, SE standard error,A p value < 0.05 was considered nominally significant.
