# Supplementary material for: Investigation of the association between circulating inflammatory proteins and encephalitis risk in Europeans by two-sample Mendelian randomization analysis
Source: Front Neurol. 2025 Feb 11;15:1450735. doi: 10.3389/fneur.2024.1450735 (PMC11850273; doi:10.3389/fneur.2024.1450735)
Supplement: Supplementary file 2 [file Presentation_1.zip › Supplementary Figure/Supplementary Figure13.pdf]

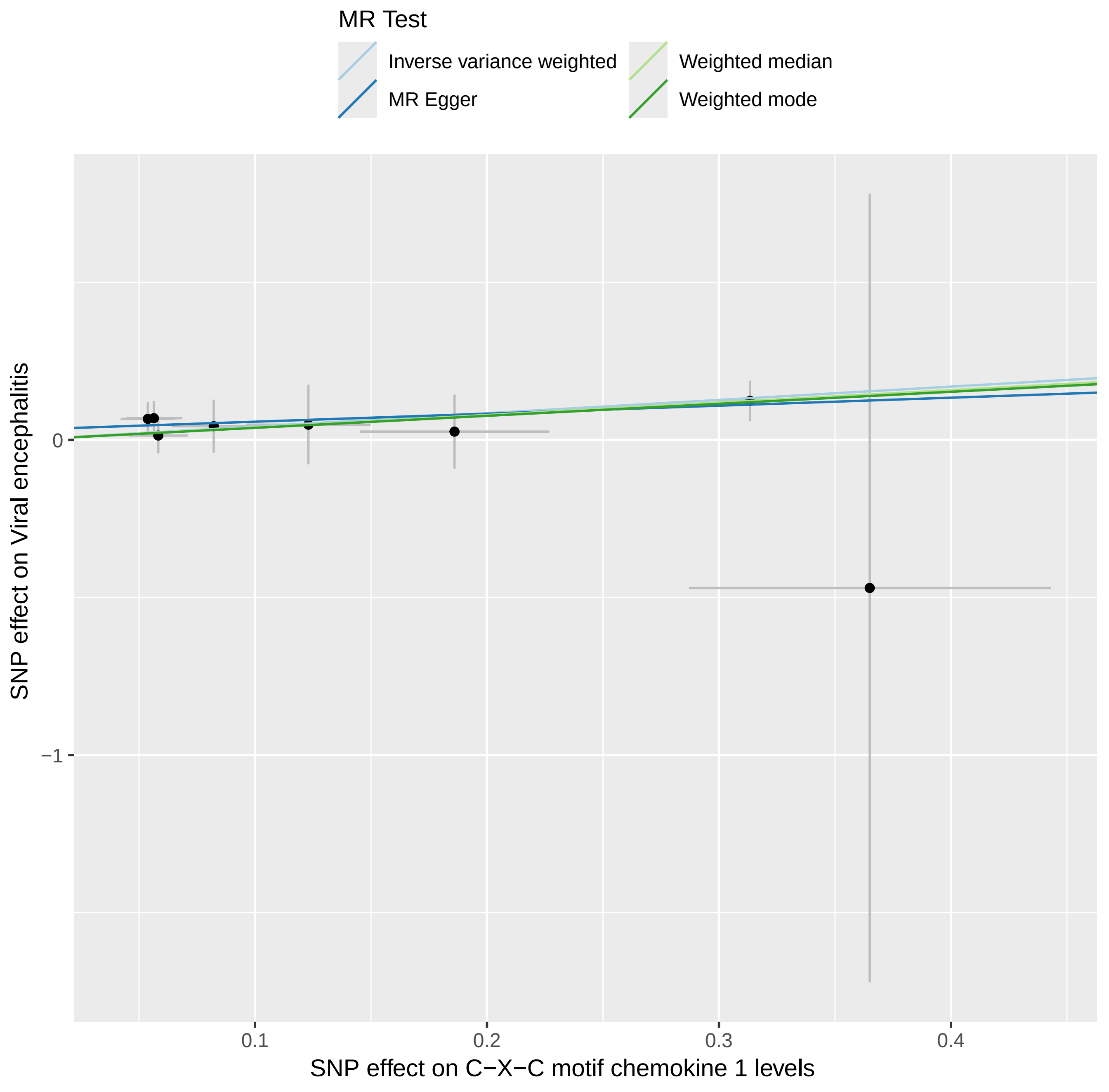

MR Test
Inverse variance weighted
MR Egger
Weighted median
Weighted mode
SNP effect on Viral encephalitis
0
−1
0.1
0.2
0.3
0.4
SNP effect on C−X−C motif chemokine 1 levels
