# Supplementary material for: Investigation of the association between circulating inflammatory proteins and encephalitis risk in Europeans by two-sample Mendelian randomization analysis
Source: Front Neurol. 2025 Feb 11;15:1450735. doi: 10.3389/fneur.2024.1450735 (PMC11850273; doi:10.3389/fneur.2024.1450735)
Supplement: Supplementary file 2 [file Presentation_1.zip › Supplementary Figure/Supplementary Figure22.pdf]

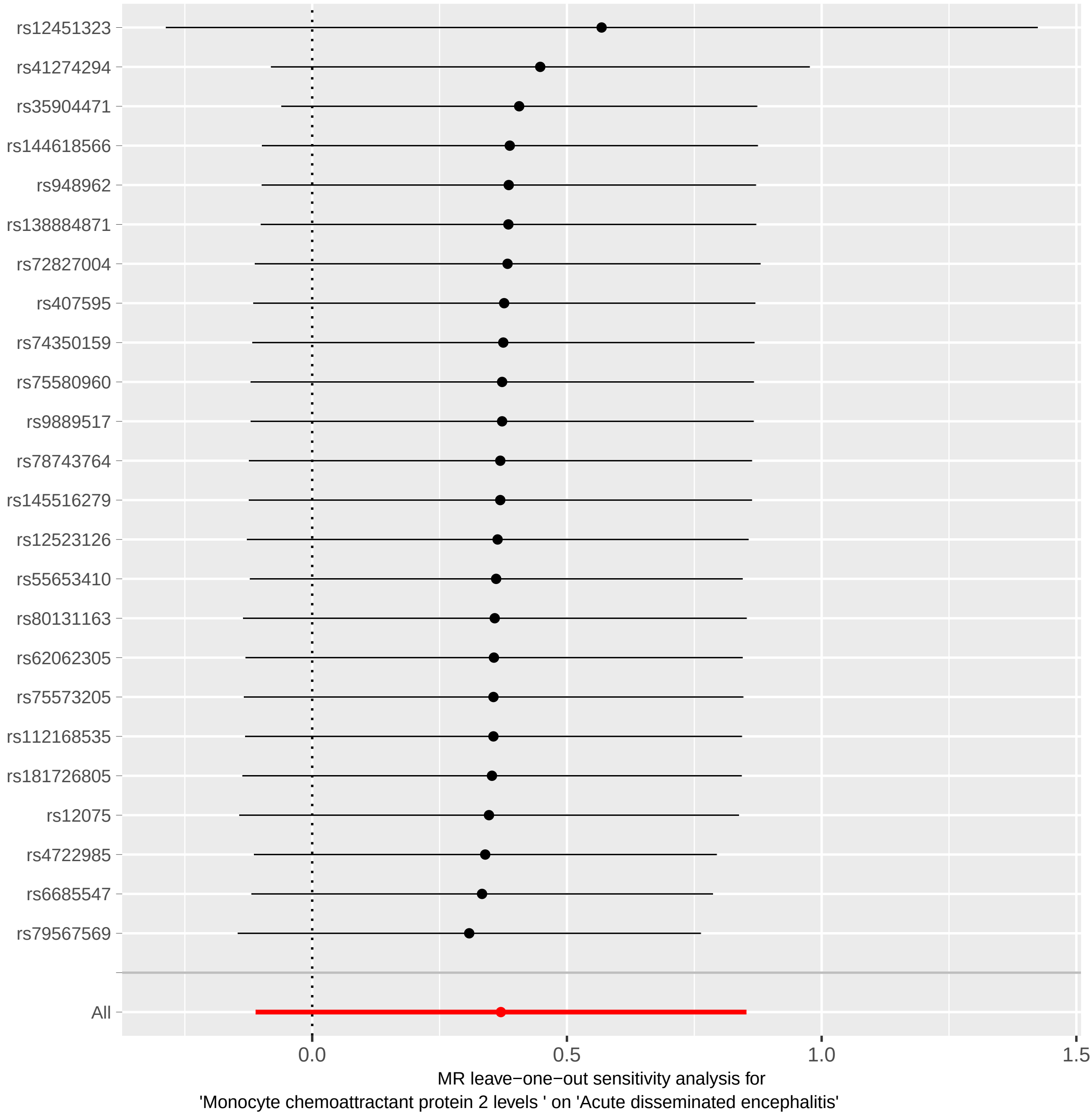

rs12451323
rs41274294
rs35904471
rs144618566
rs948962
rs138884871
rs72827004
rs407595
rs74350159
rs75580960
rs9889517
rs78743764
rs145516279
rs12523126
rs55653410
rs80131163
rs62062305
rs75573205
rs112168535
rs181726805
rs12075
rs4722985
rs6685547
rs79567569
All
0.0
0.5
1.0
1.5
MR leave−one−out sensitivity analysis for
'Monocyte chemoattractant protein 2 levels ' on 'Acute disseminated encephalitis'
