# Supplementary figures and images for: Investigation of the association between circulating inflammatory proteins and encephalitis risk in Europeans by two-sample Mendelian randomization analysis
Source: Front Neurol. 2025 Feb 11;15:1450735. doi: 10.3389/fneur.2024.1450735 (PMC11850273; doi:10.3389/fneur.2024.1450735)

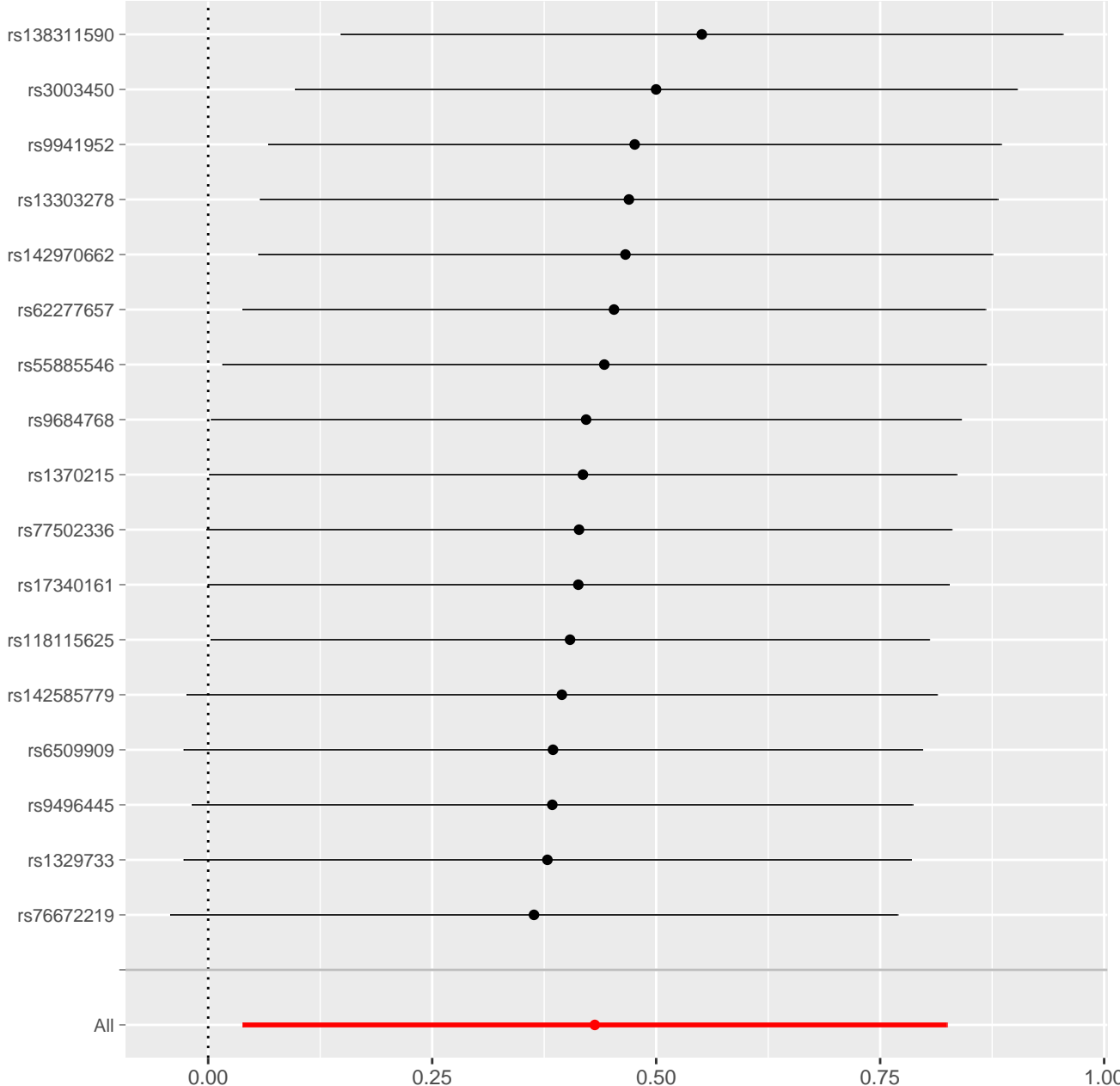

MR leave-one-out sensitivity analysis for  
'Artemin levels' on 'Viral encephalitis'

Supplement: Supplementary file 2 [file Presentation_1.zip › Supplementary Figure/Supplementary Figure1.pdf]

# MR Method

- Inverse variance weighted
- MR Egger

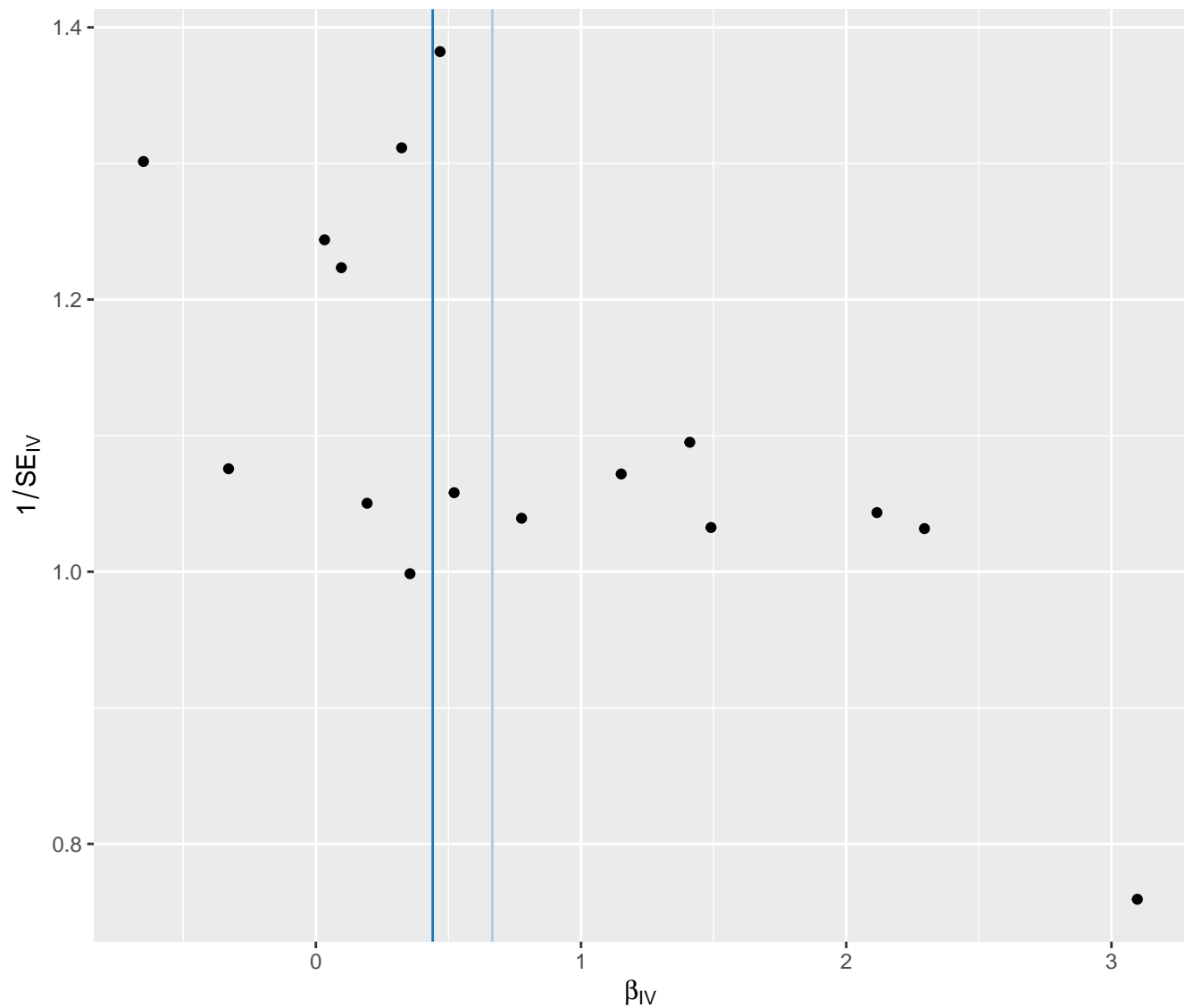

Supplement: Supplementary file 2 [file Presentation_1.zip › Supplementary Figure/Supplementary Figure10.pdf]

# MR Test

- Inverse variance weighted
- MR Egger
- Weighted median
- Weighted mode

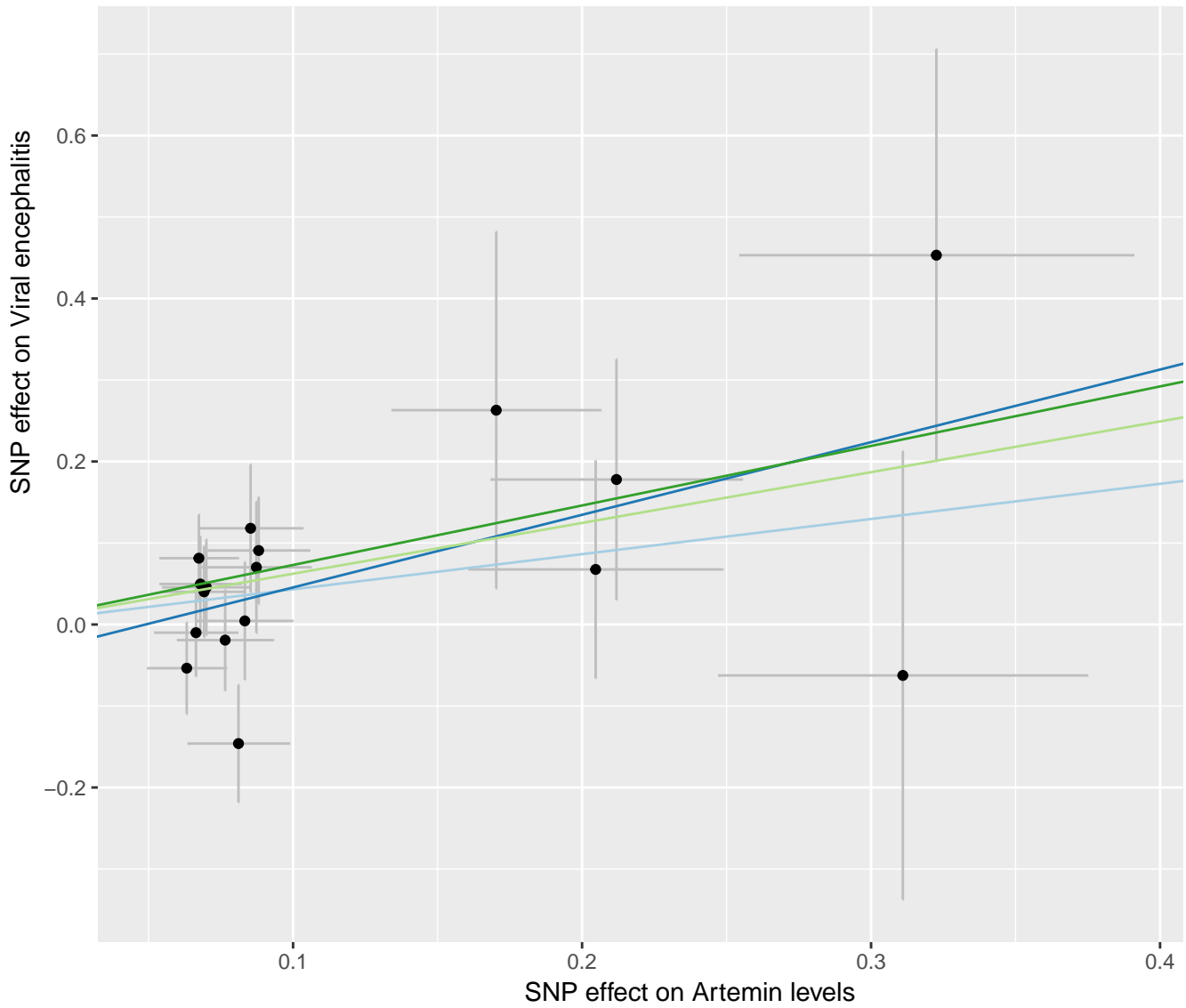

Supplement: Supplementary file 2 [file Presentation_1.zip › Supplementary Figure/Supplementary Figure11.pdf]

# MR Test

- Inverse variance weighted
- MR Egger
- Weighted median
- Weighted mode

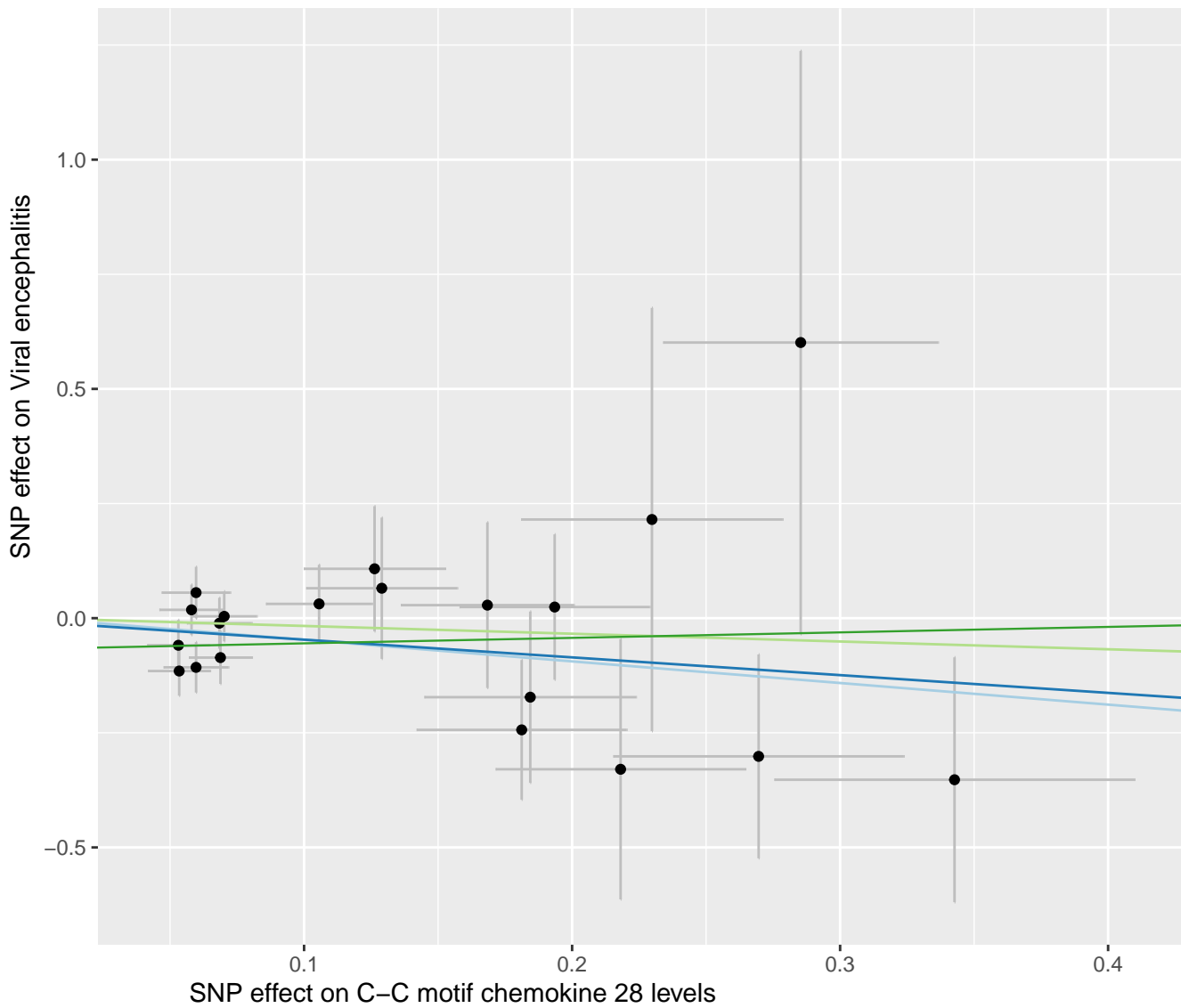

Supplement: Supplementary file 2 [file Presentation_1.zip › Supplementary Figure/Supplementary Figure12.pdf]

# MR Test

- Inverse variance weighted
- MR Egger
- Weighted median
- Weighted mode

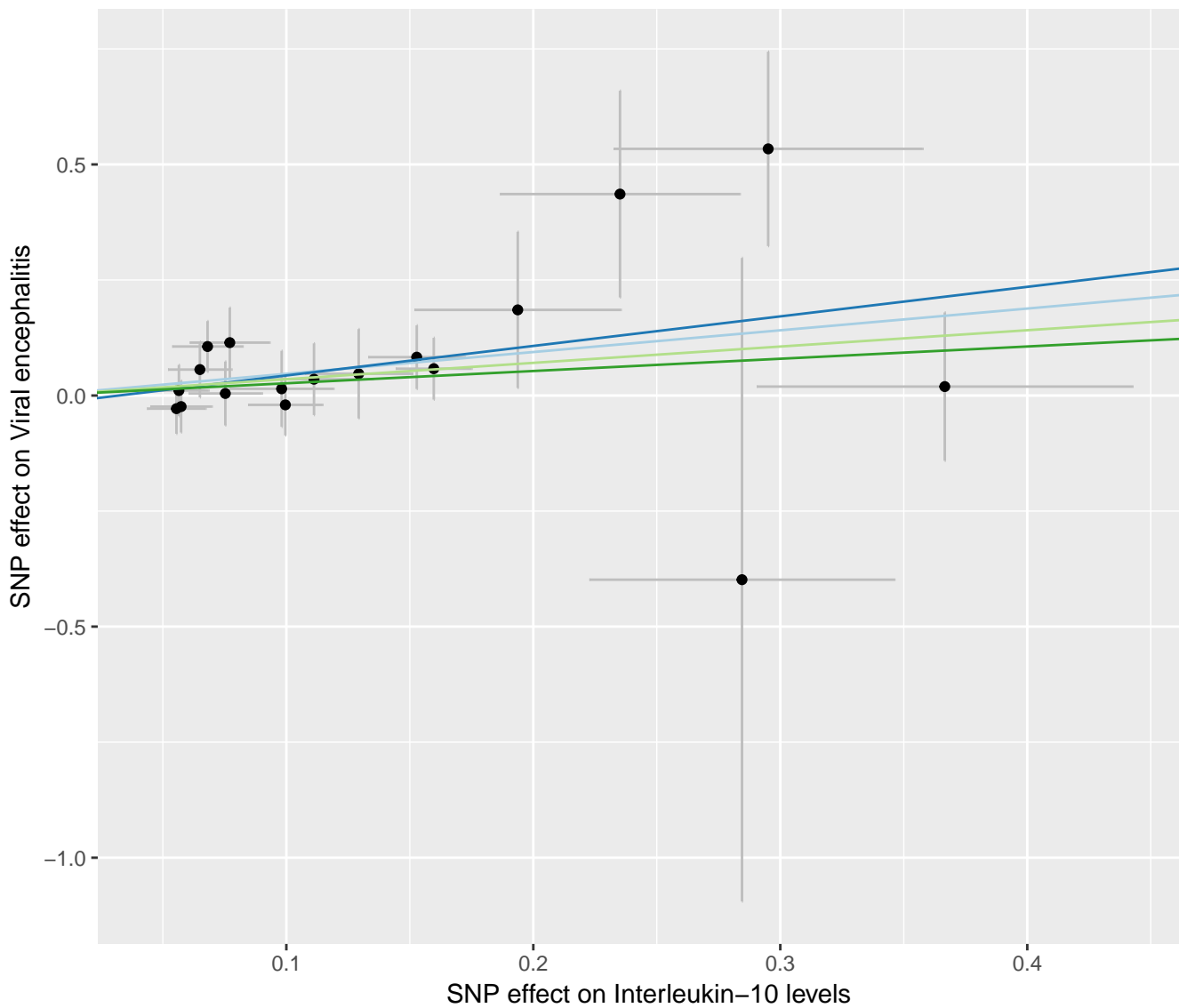

Supplement: Supplementary file 2 [file Presentation_1.zip › Supplementary Figure/Supplementary Figure14.pdf]

# MR Test

- Inverse variance weighted
- MR Egger
- Weighted median
- Weighted mode

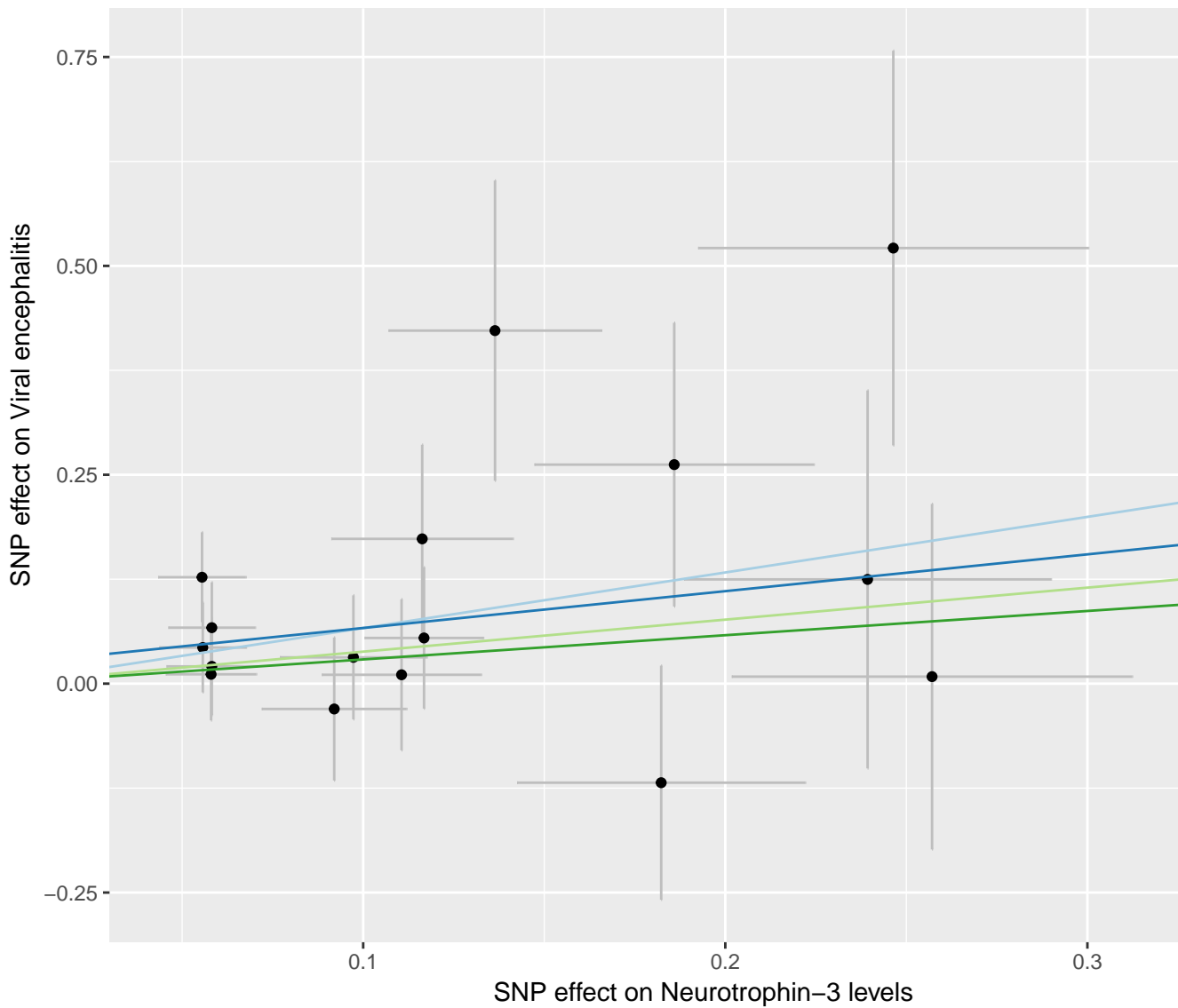

Supplement: Supplementary file 2 [file Presentation_1.zip › Supplementary Figure/Supplementary Figure15.pdf]

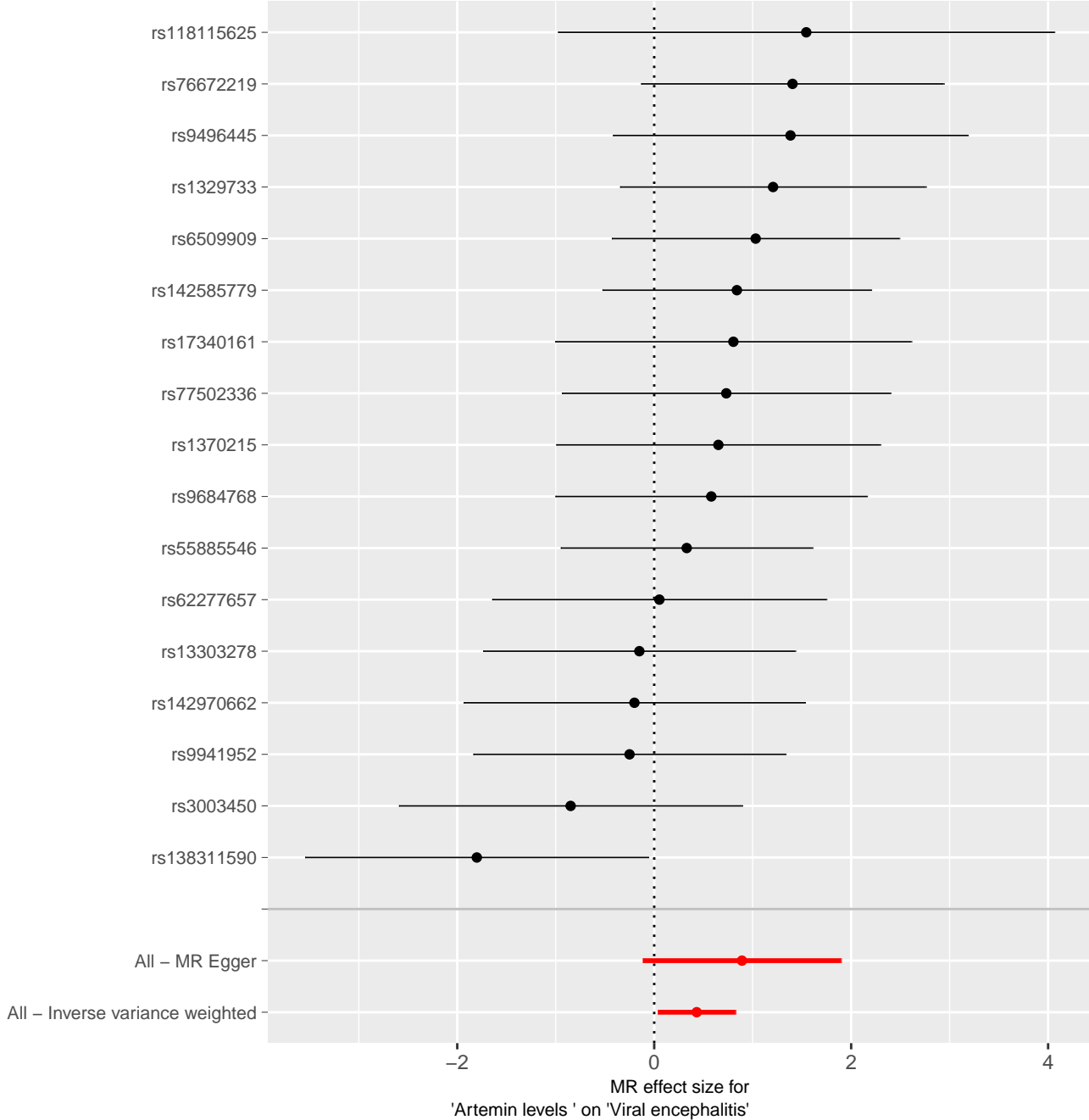

Supplement: Supplementary file 2 [file Presentation_1.zip › Supplementary Figure/Supplementary Figure16.pdf]

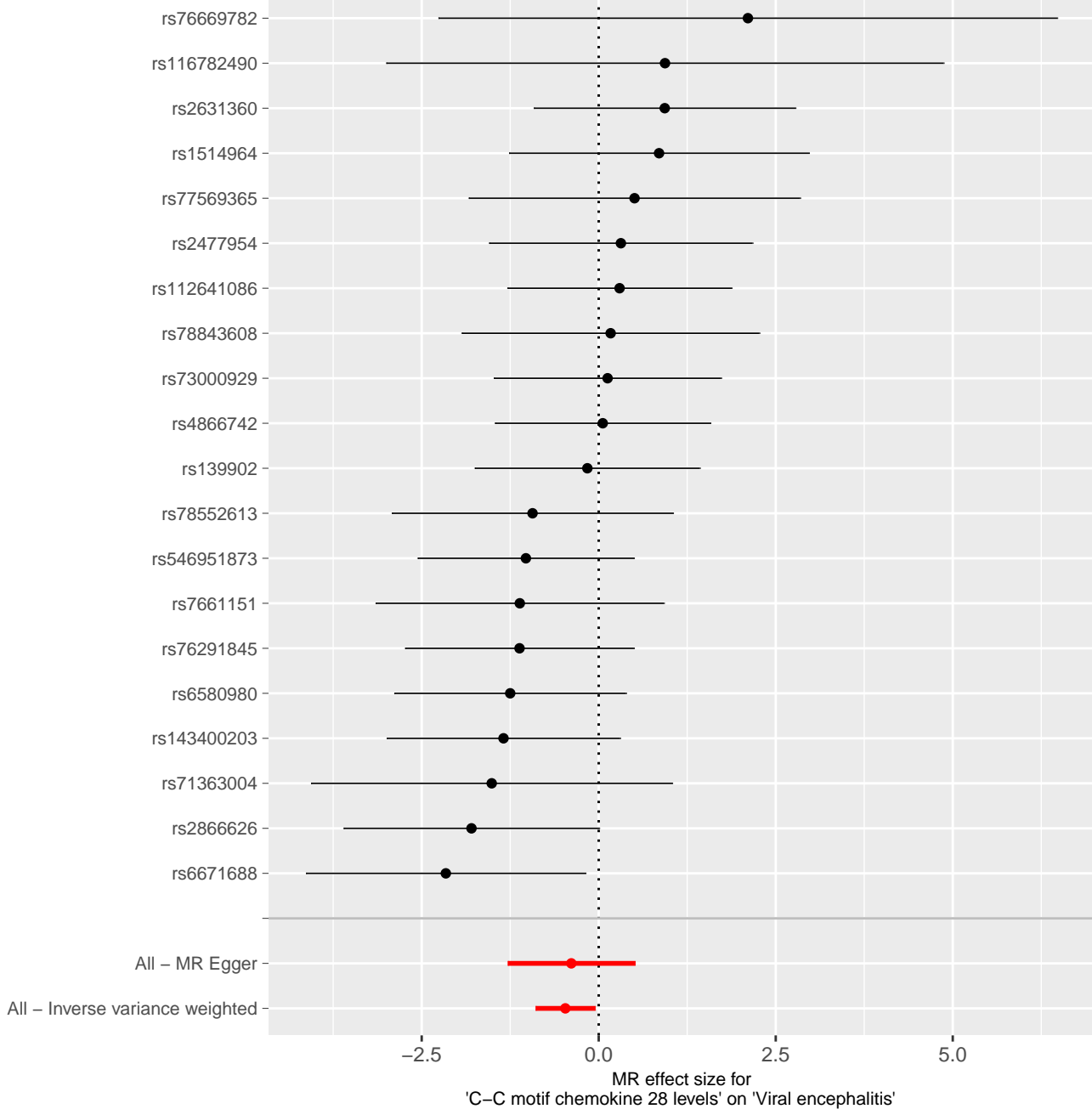

Supplement: Supplementary file 2 [file Presentation_1.zip › Supplementary Figure/Supplementary Figure17.pdf]

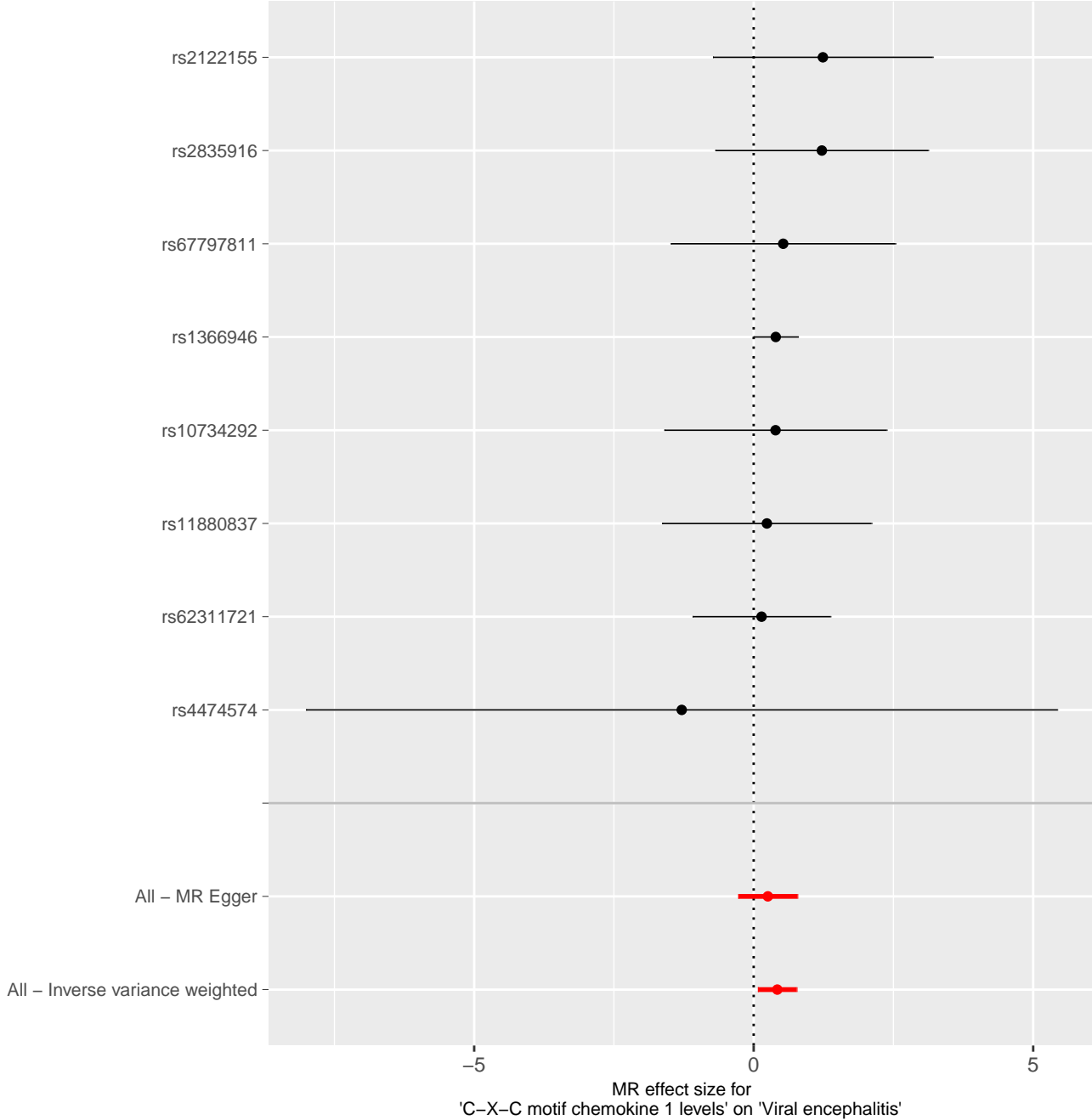

Supplement: Supplementary file 2 [file Presentation_1.zip › Supplementary Figure/Supplementary Figure18.pdf]

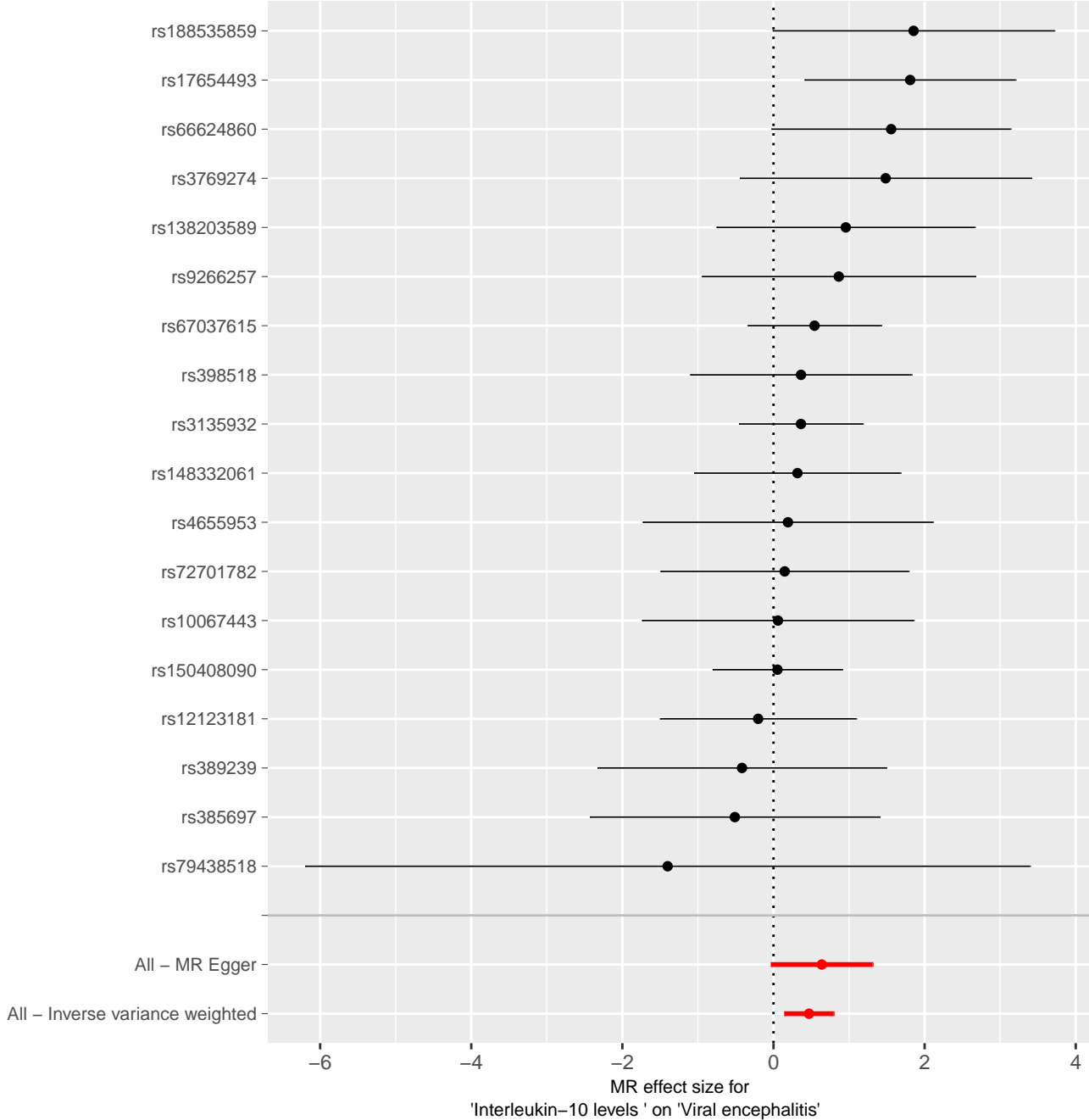

Supplement: Supplementary file 2 [file Presentation_1.zip › Supplementary Figure/Supplementary Figure19.pdf]

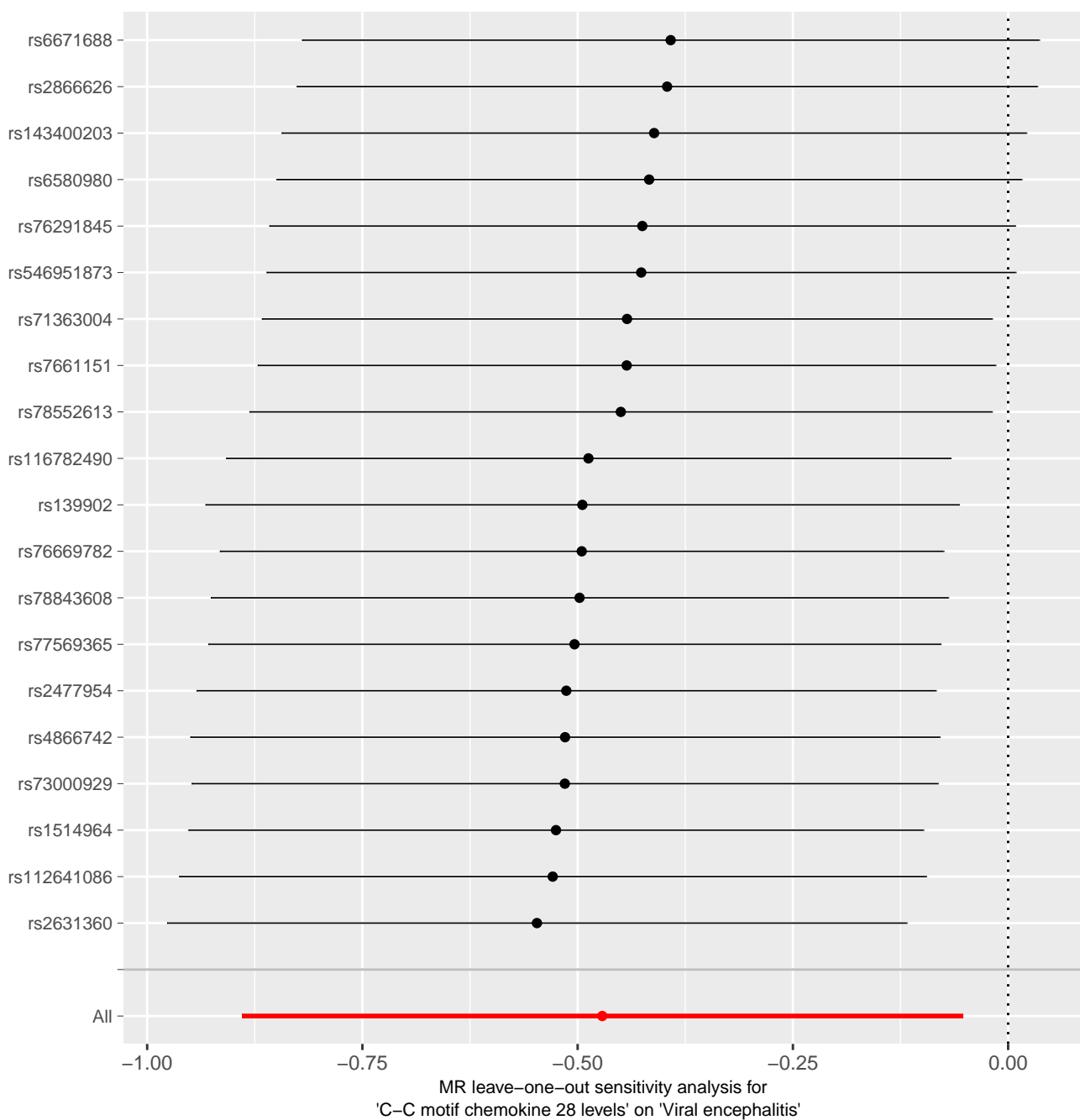

Supplement: Supplementary file 2 [file Presentation_1.zip › Supplementary Figure/Supplementary Figure2.pdf]

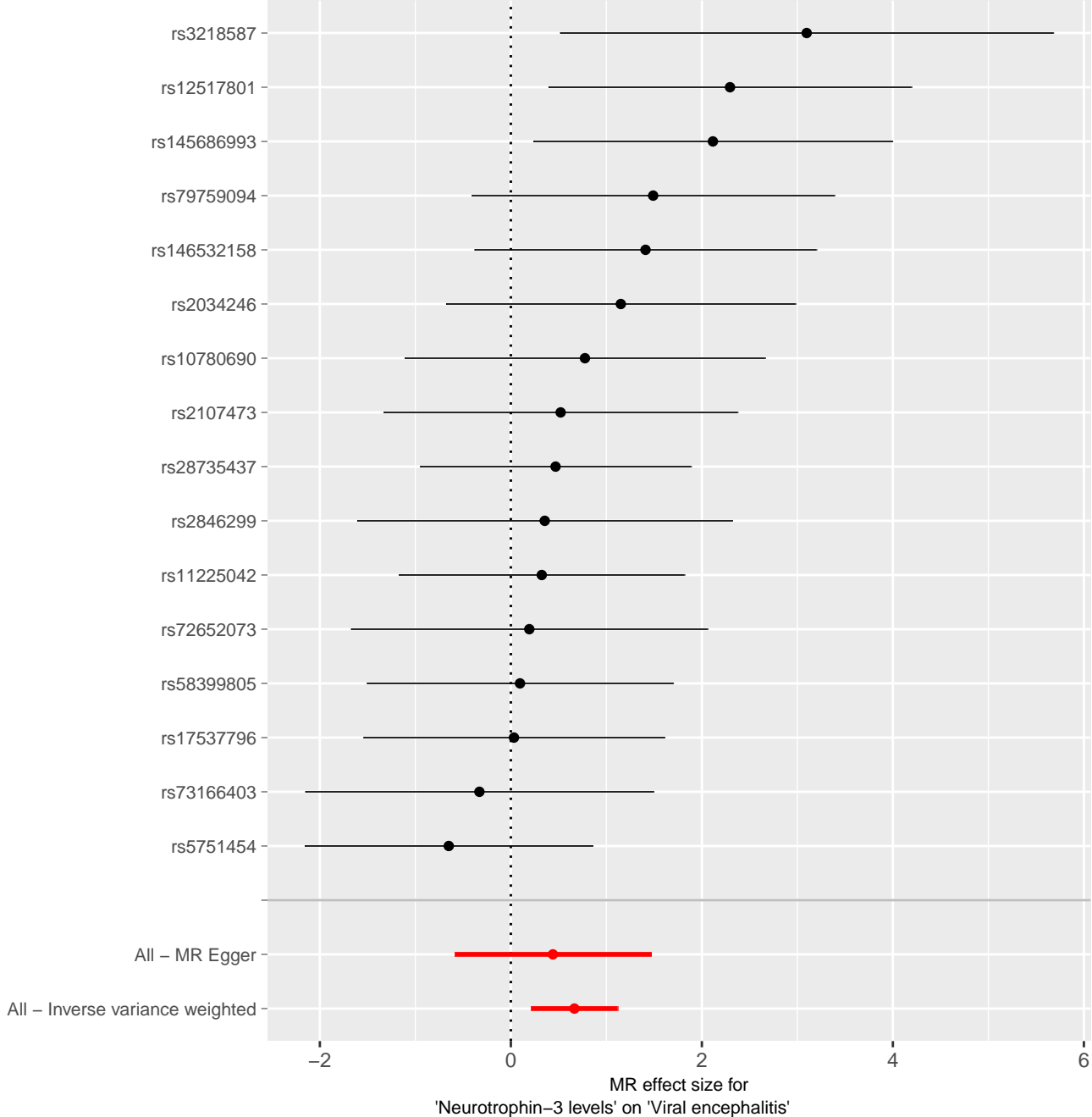

Supplement: Supplementary file 2 [file Presentation_1.zip › Supplementary Figure/Supplementary Figure20.pdf]

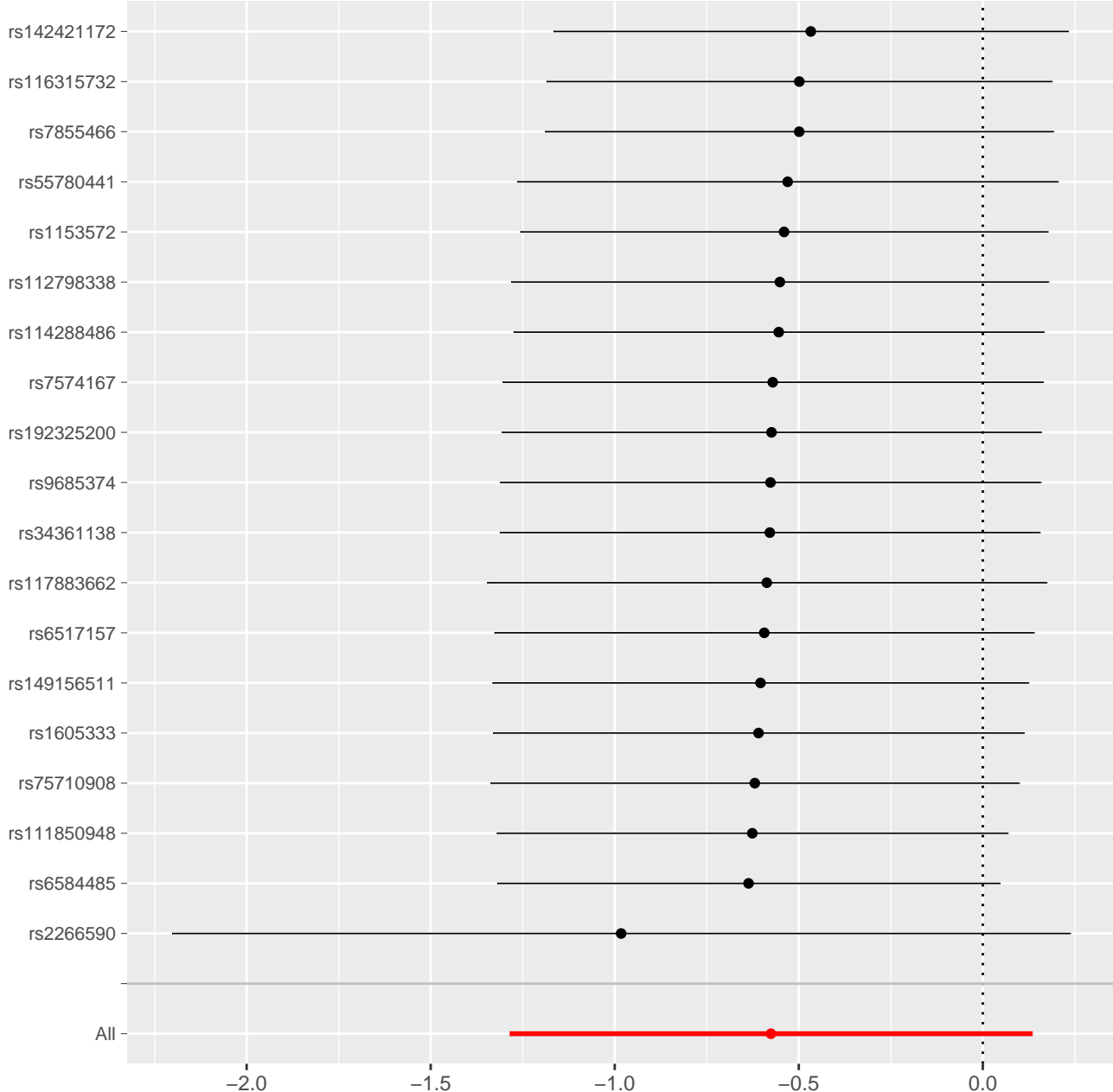

Supplement: Supplementary file 2 [file Presentation_1.zip › Supplementary Figure/Supplementary Figure21.pdf]

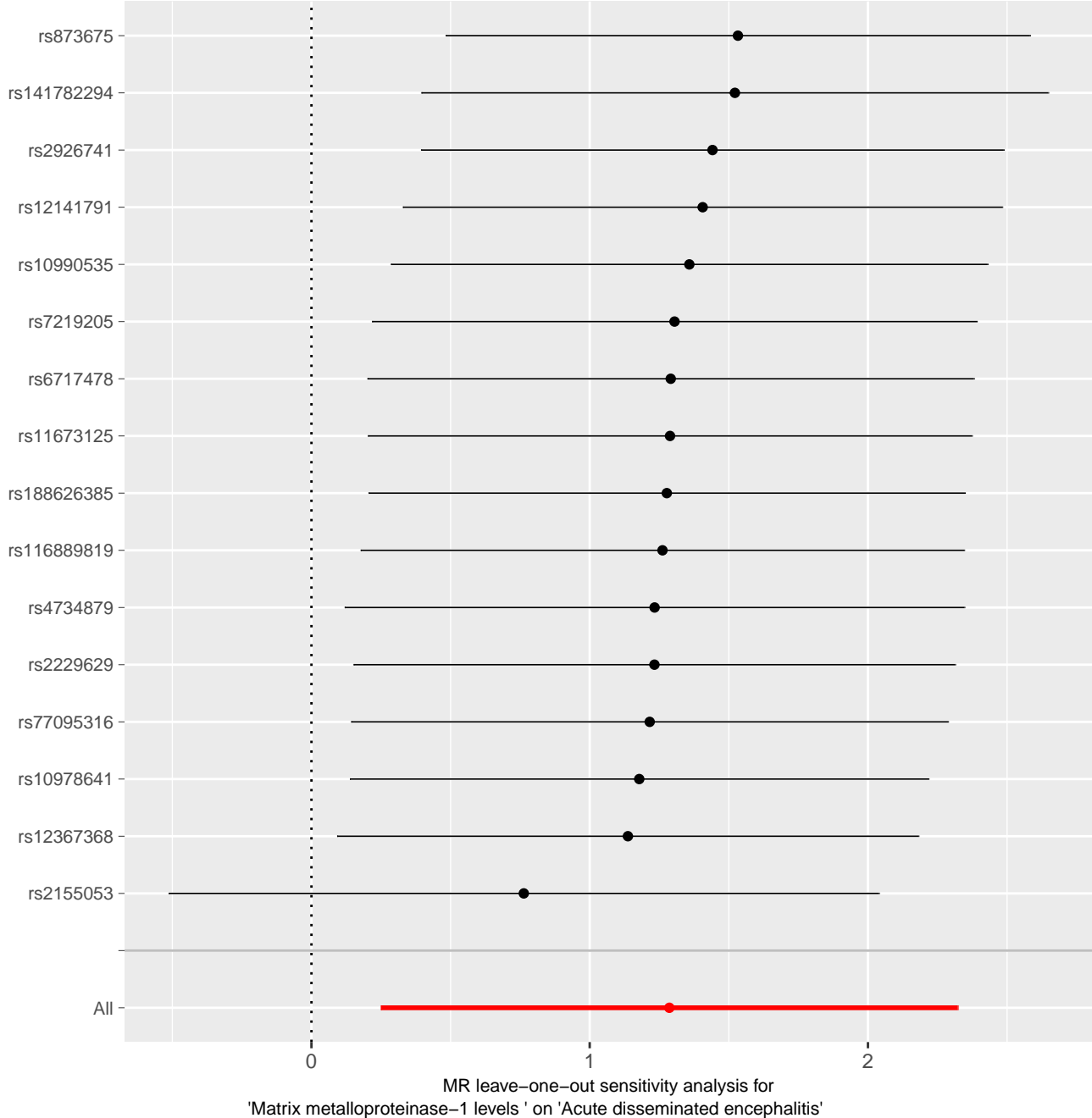

Supplement: Supplementary file 2 [file Presentation_1.zip › Supplementary Figure/Supplementary Figure23.pdf]

# MR Method

- Inverse variance weighted
- MR Egger

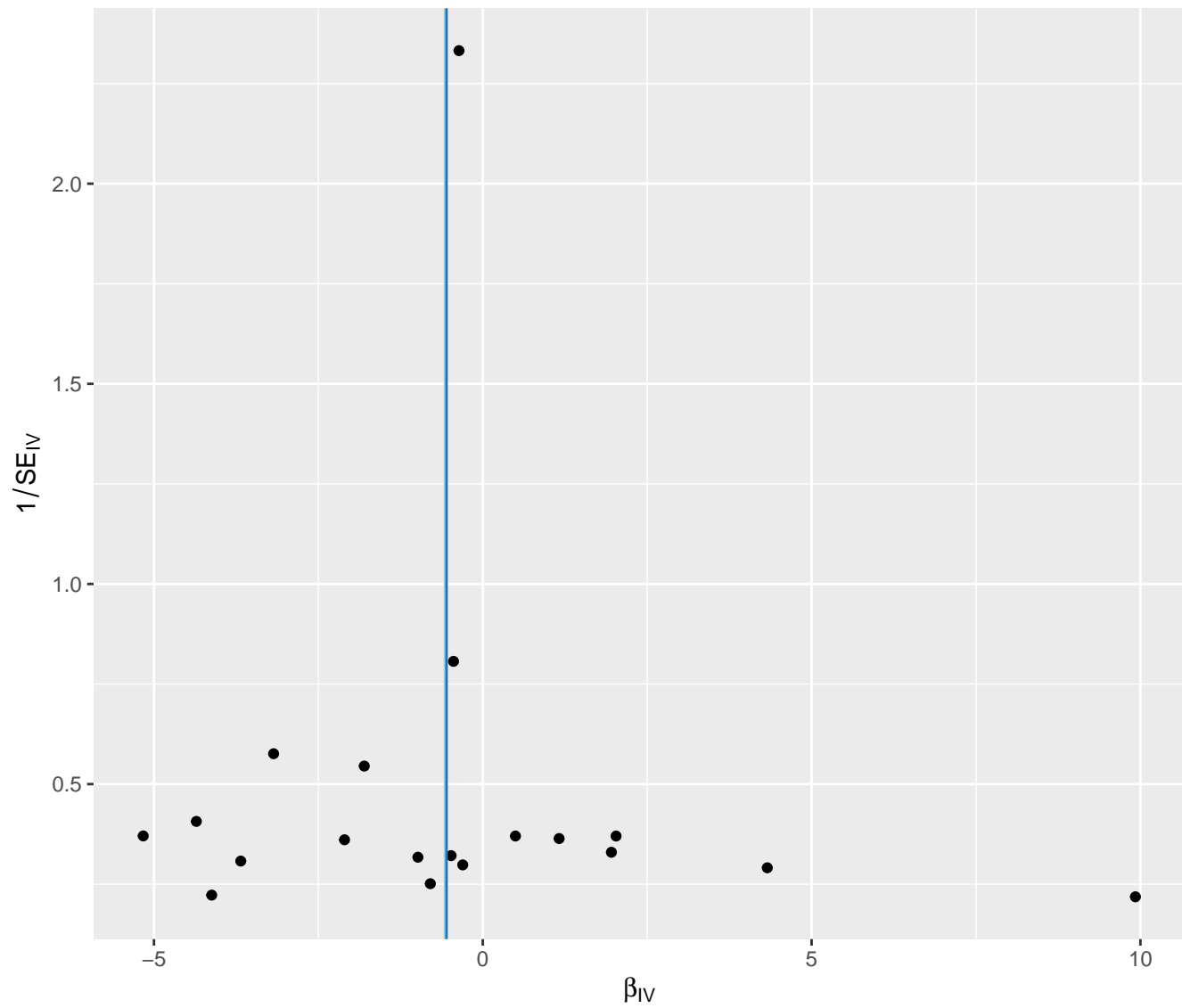

Supplement: Supplementary file 2 [file Presentation_1.zip › Supplementary Figure/Supplementary Figure24.pdf]

# MR Method

- Inverse variance weighted
- MR Egger

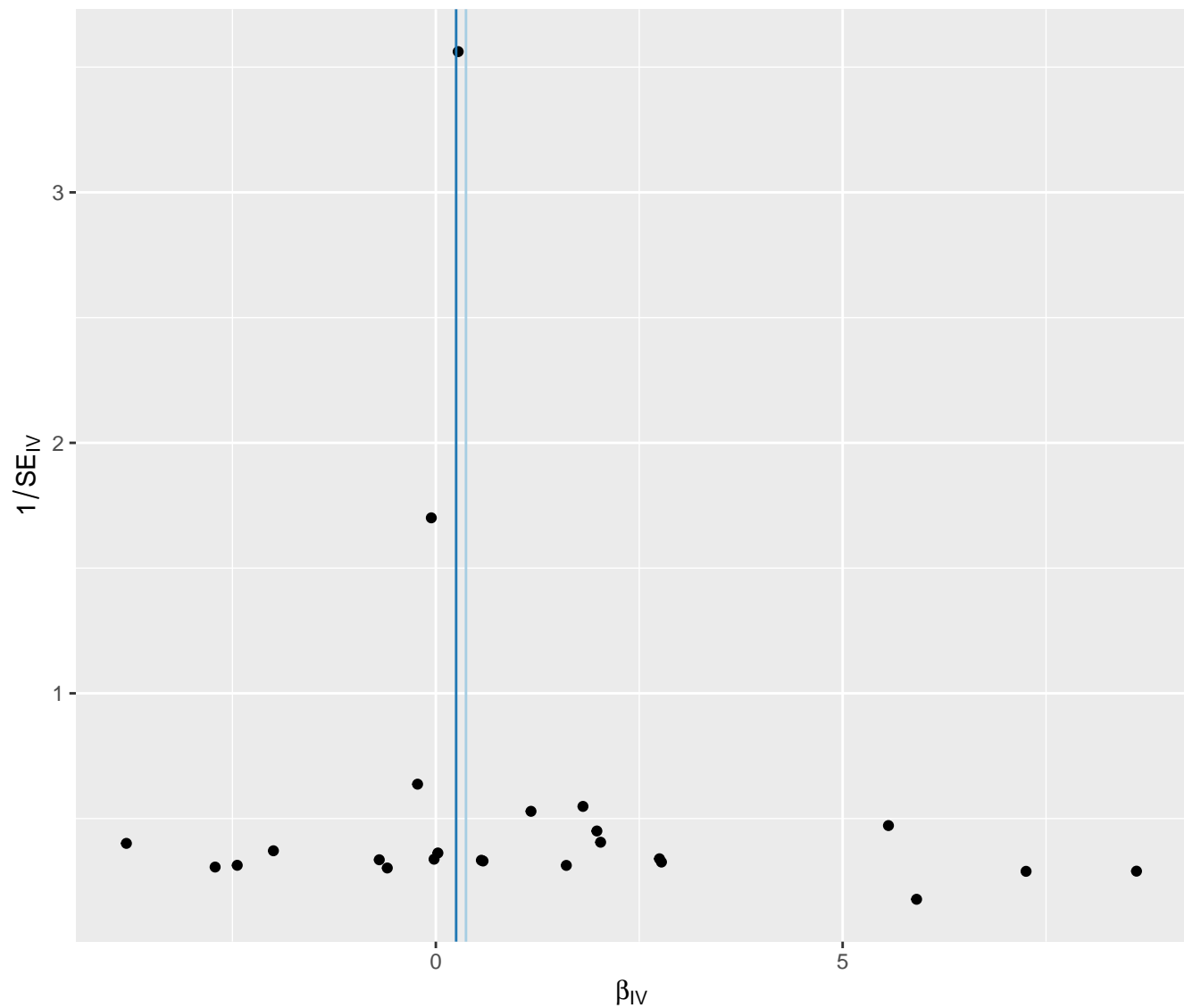

Supplement: Supplementary file 2 [file Presentation_1.zip › Supplementary Figure/Supplementary Figure25.pdf]

# MR Method

- Inverse variance weighted
- MR Egger

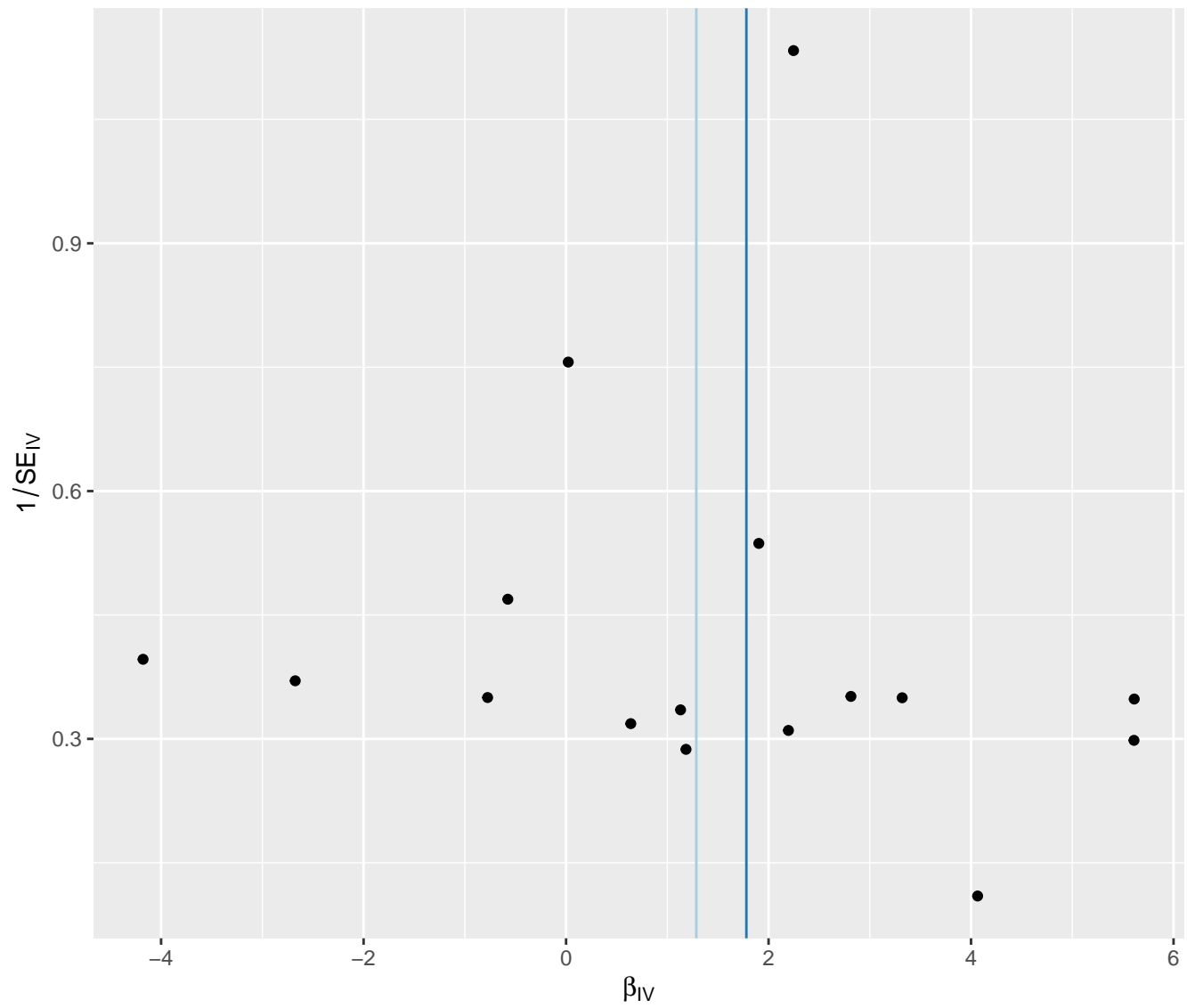

Supplement: Supplementary file 2 [file Presentation_1.zip › Supplementary Figure/Supplementary Figure26.pdf]

# MR Test

- Inverse variance weighted
- MR Egger
- Weighted median
- Weighted mode

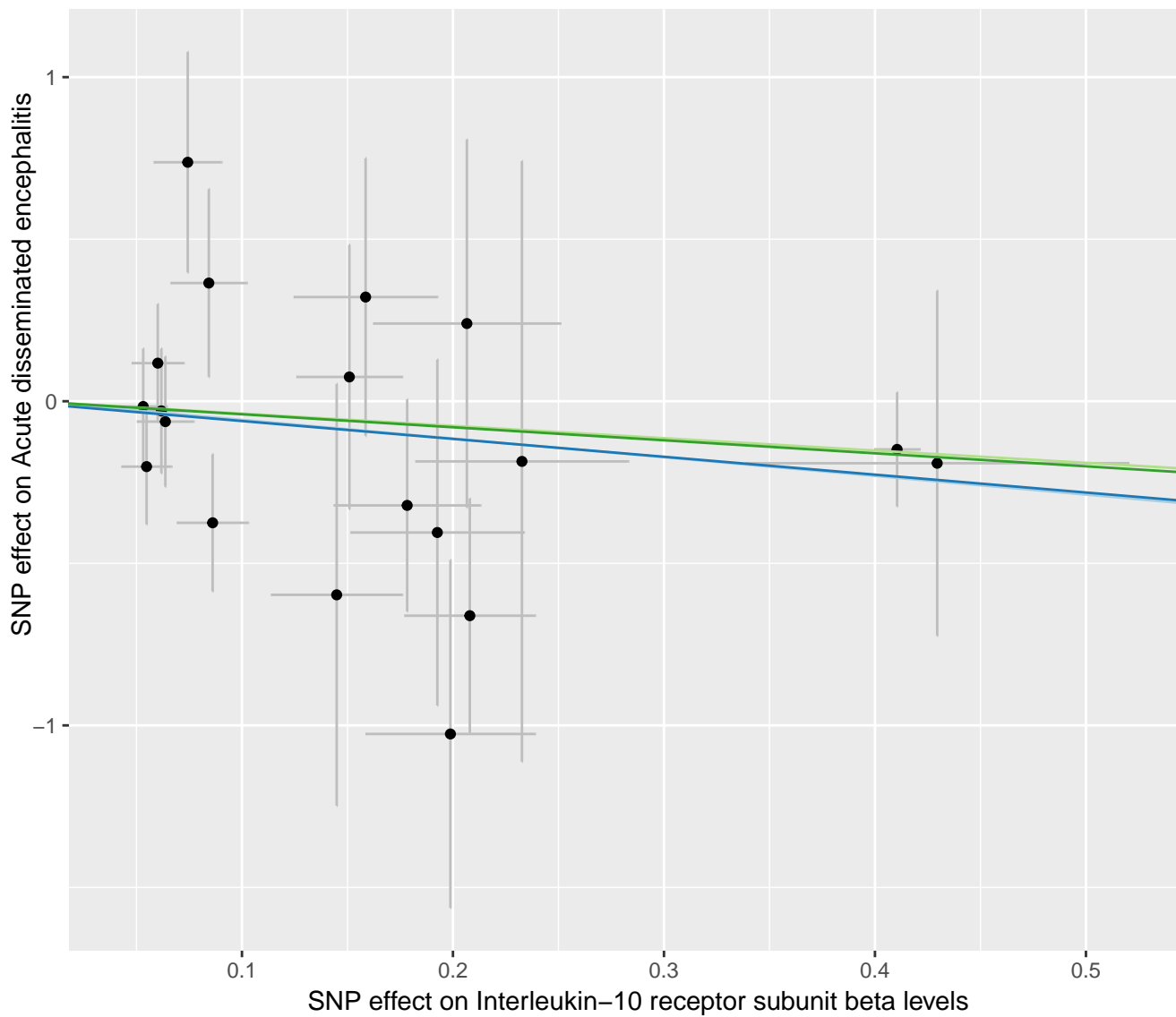

Supplement: Supplementary file 2 [file Presentation_1.zip › Supplementary Figure/Supplementary Figure27.pdf]

# MR Test

- Inverse variance weighted
- MR Egger
- Weighted median
- Weighted mode

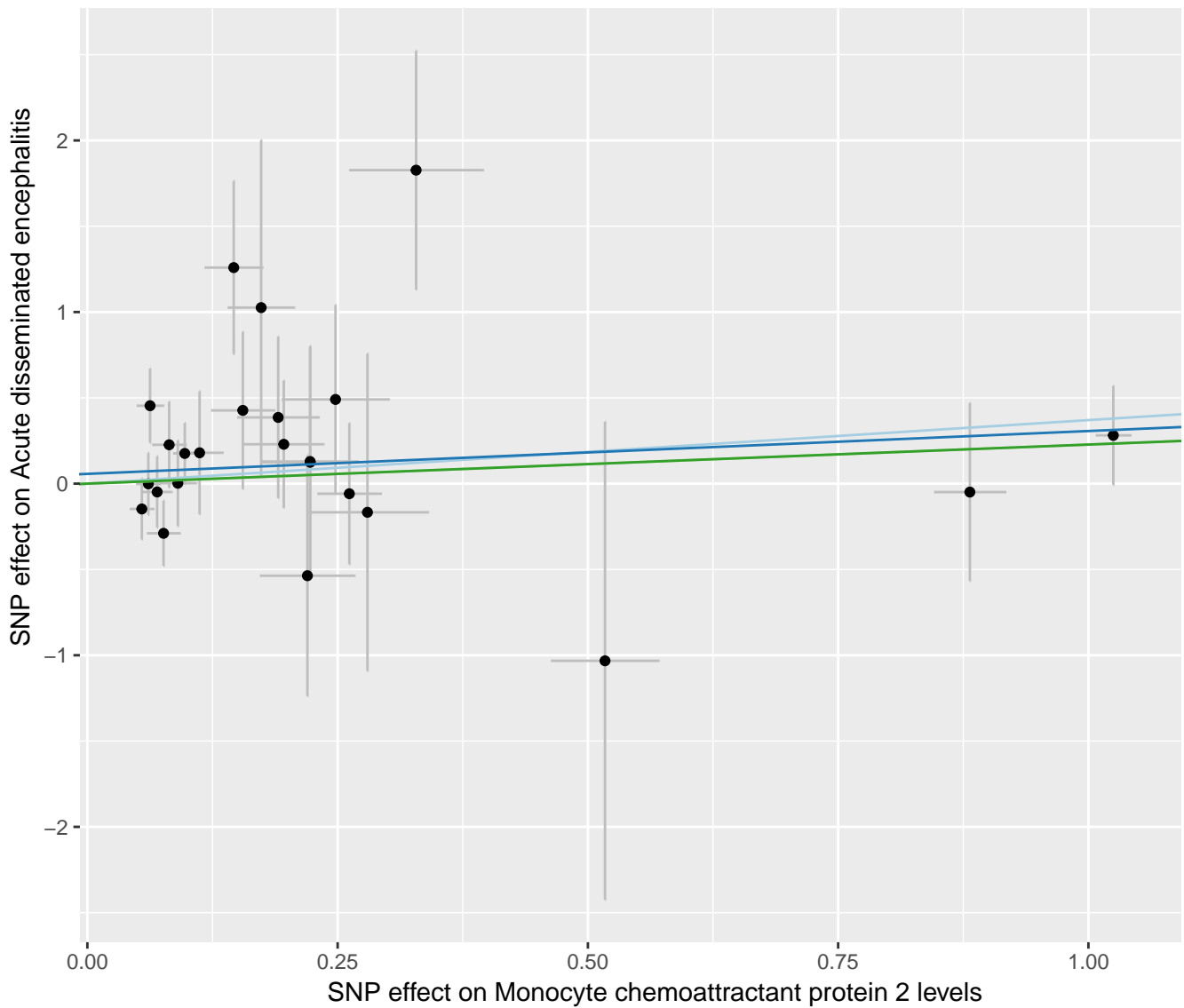

Supplement: Supplementary file 2 [file Presentation_1.zip › Supplementary Figure/Supplementary Figure28.pdf]

# MR Test

- Inverse variance weighted
- MR Egger
- Weighted median
- Weighted mode

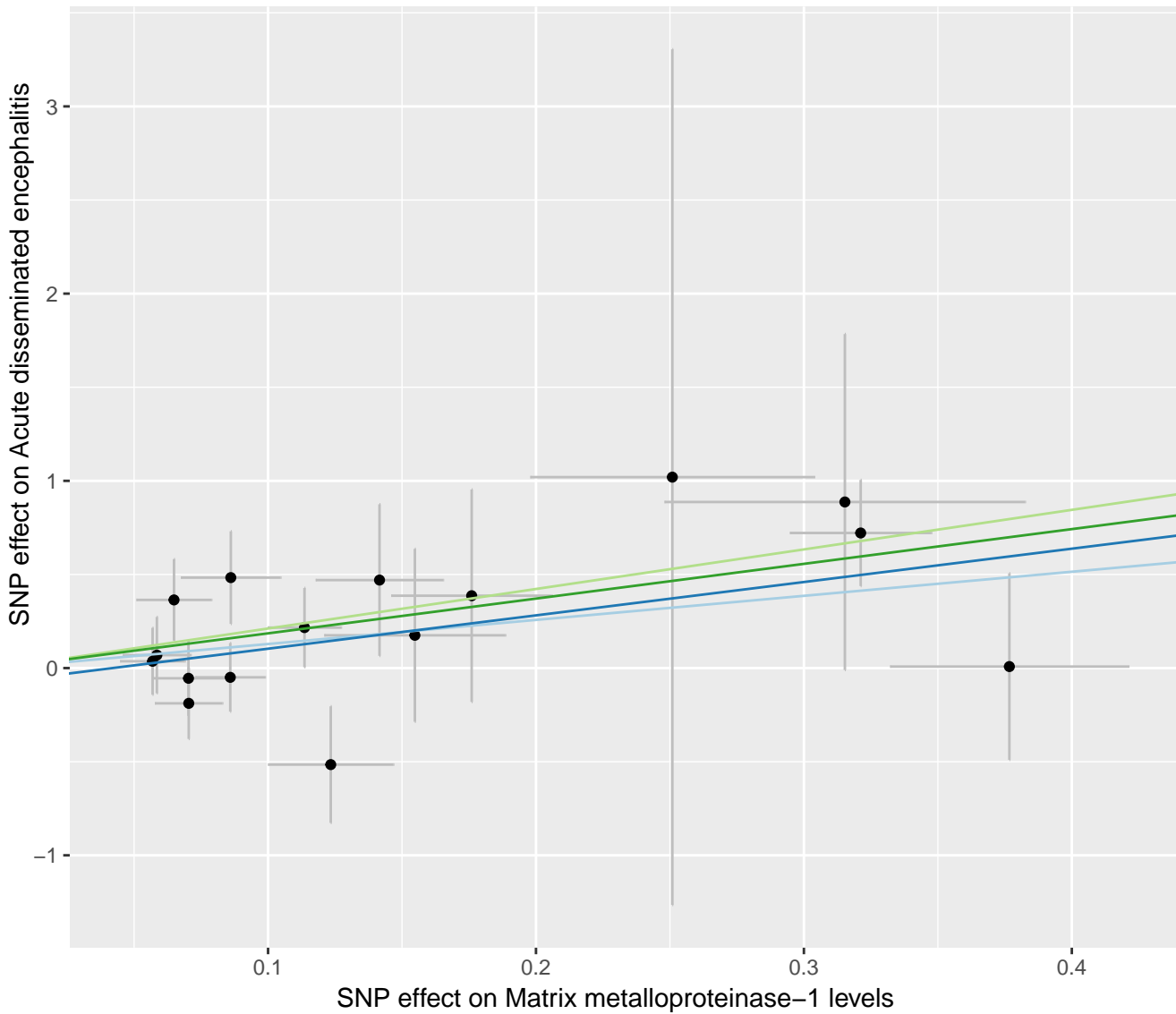

Supplement: Supplementary file 2 [file Presentation_1.zip › Supplementary Figure/Supplementary Figure29.pdf]

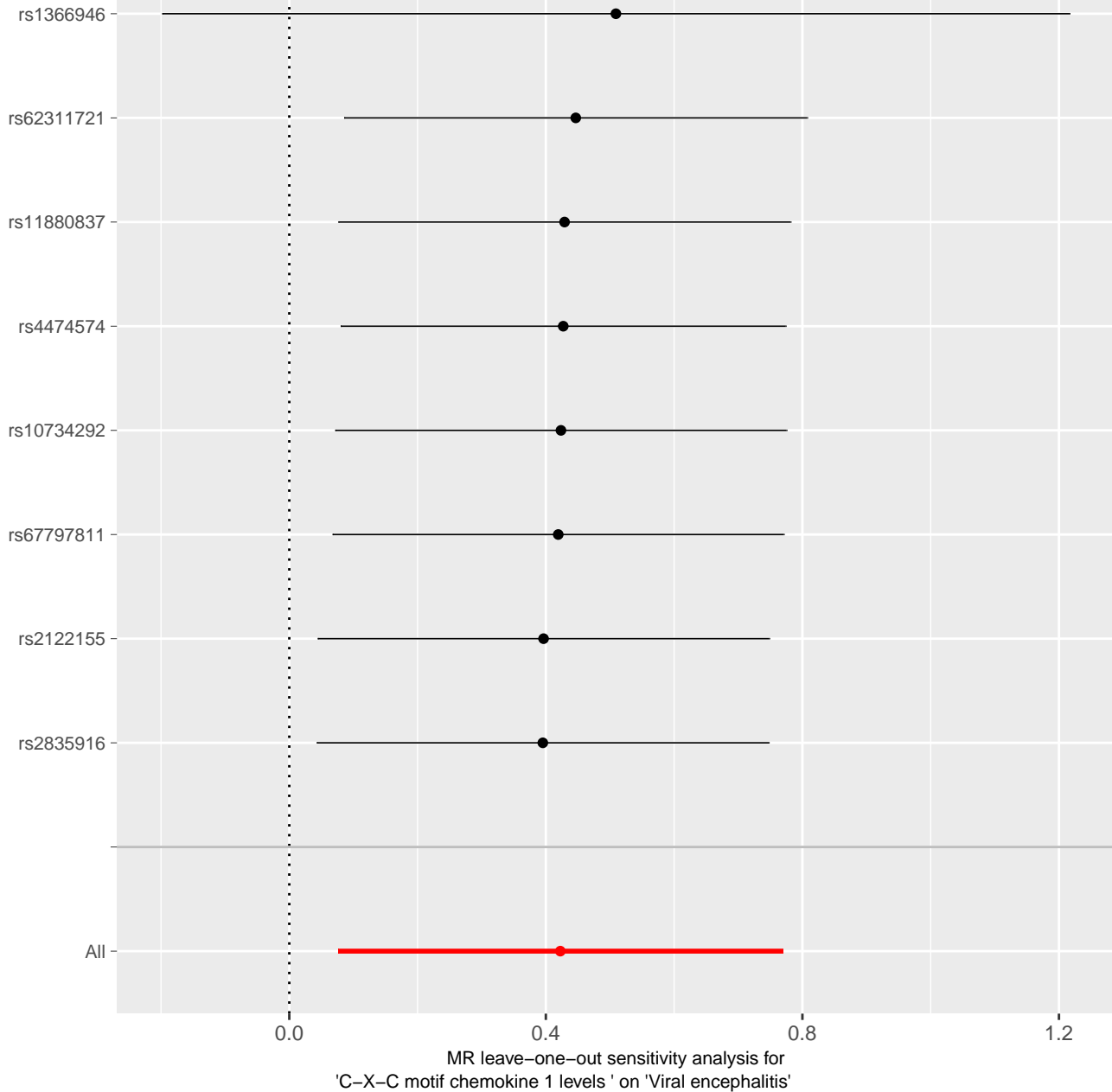

Supplement: Supplementary file 2 [file Presentation_1.zip › Supplementary Figure/Supplementary Figure3.pdf]

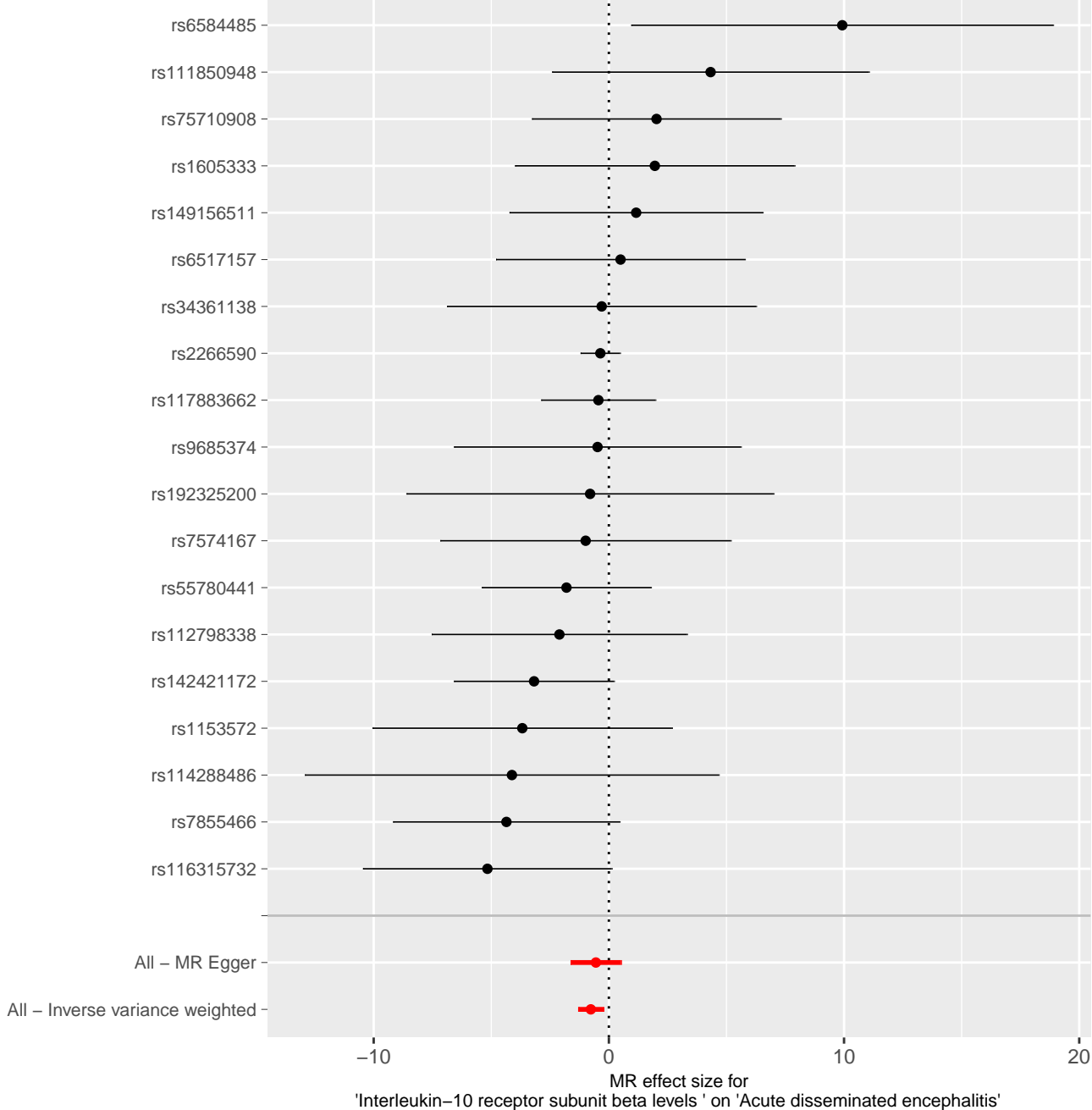

Supplement: Supplementary file 2 [file Presentation_1.zip › Supplementary Figure/Supplementary Figure30.pdf]

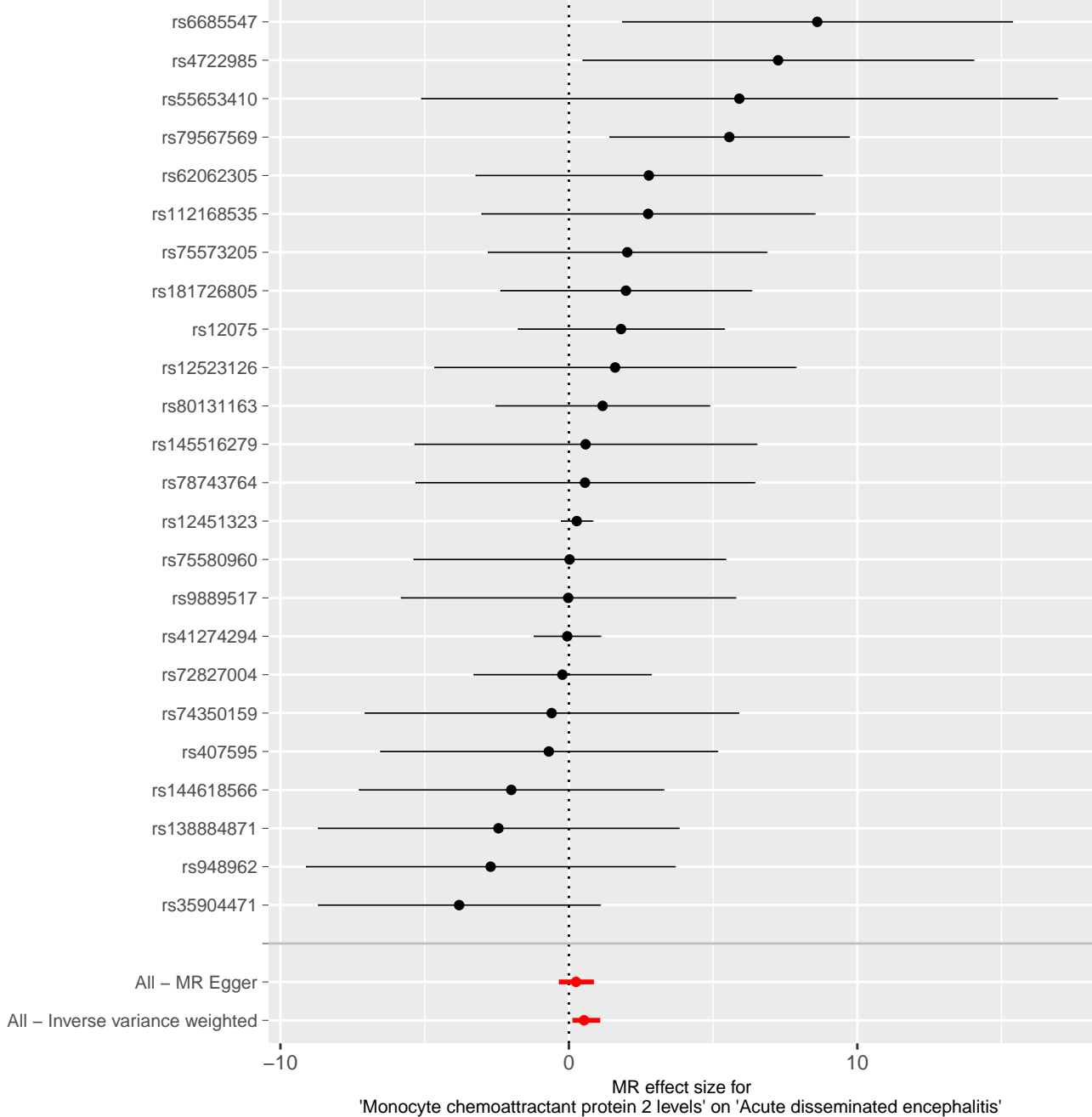

Supplement: Supplementary file 2 [file Presentation_1.zip › Supplementary Figure/Supplementary Figure31.pdf]

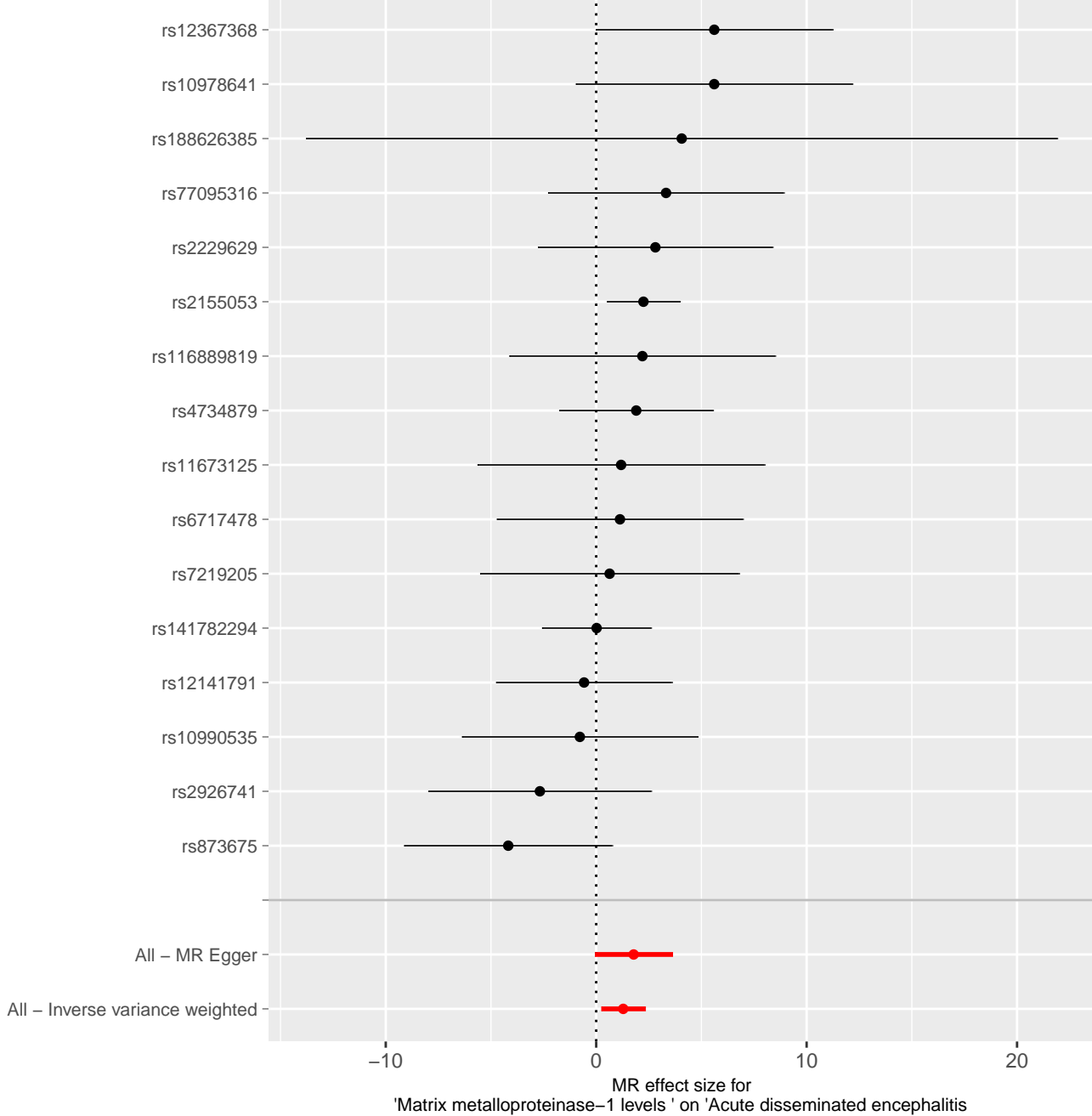

Supplement: Supplementary file 2 [file Presentation_1.zip › Supplementary Figure/Supplementary Figure32.pdf]

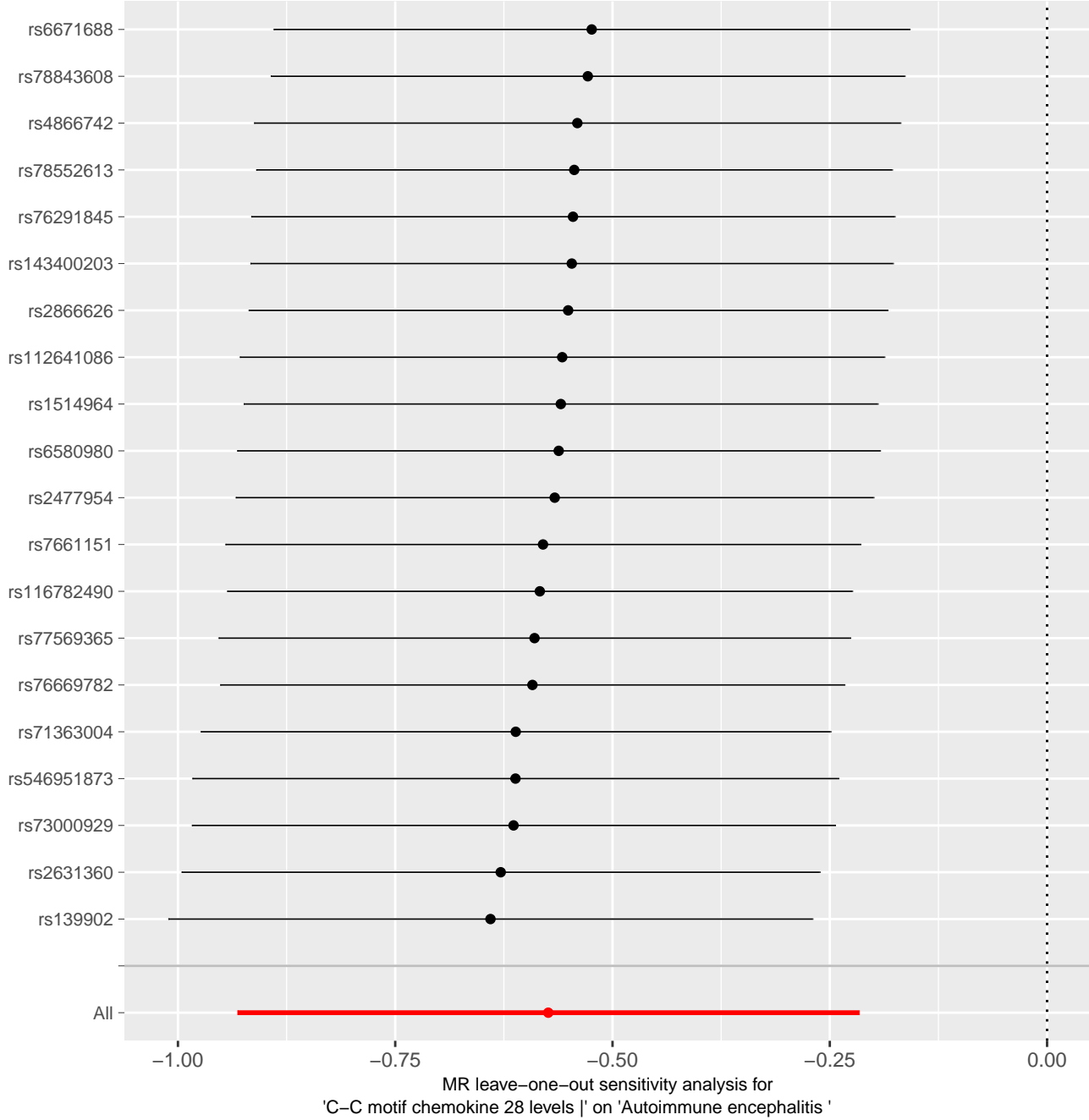

Supplement: Supplementary file 2 [file Presentation_1.zip › Supplementary Figure/Supplementary Figure33.pdf]

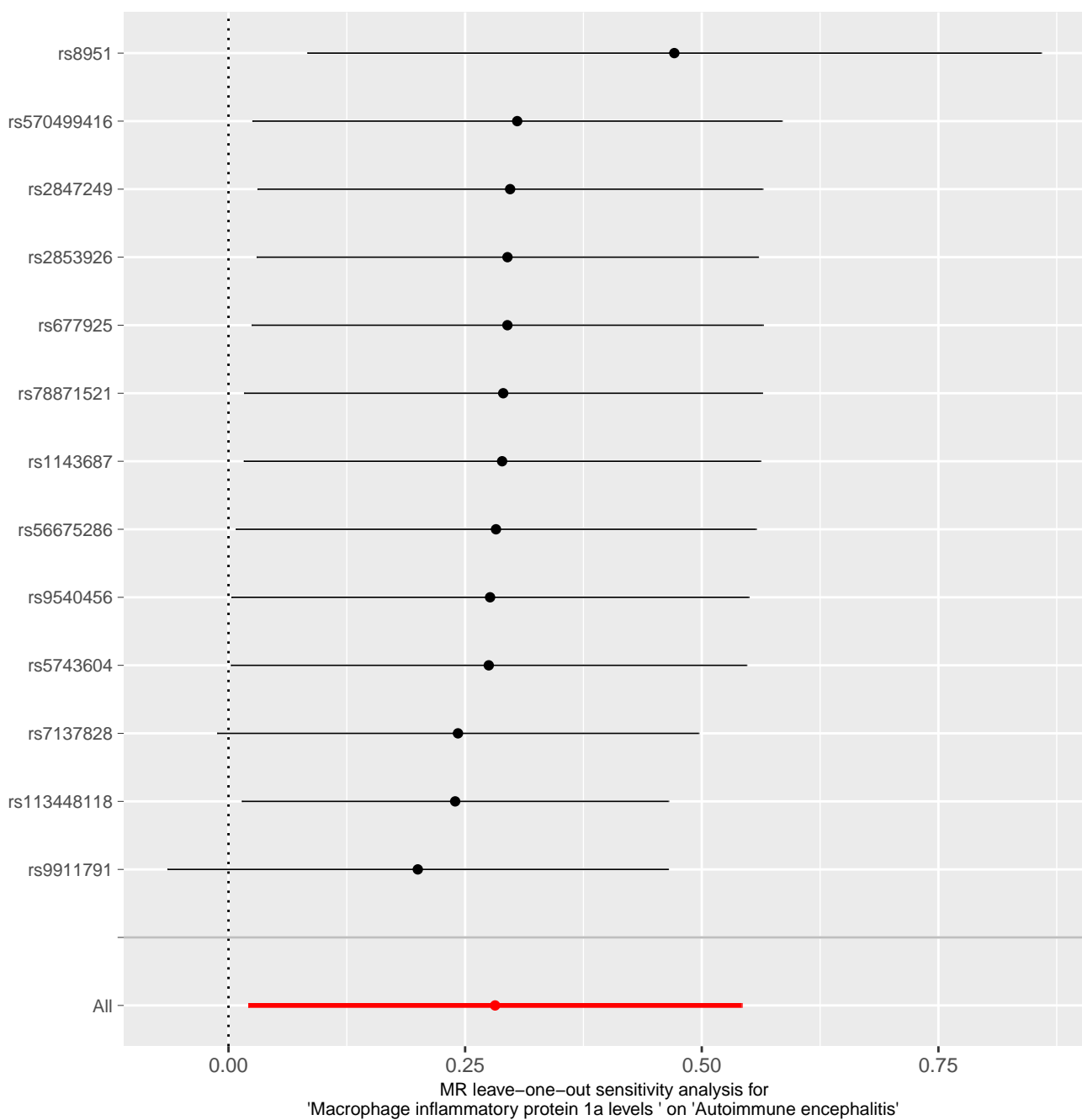

Supplement: Supplementary file 2 [file Presentation_1.zip › Supplementary Figure/Supplementary Figure34.pdf]

# MR Method

Inverse variance weighted

MR Egger

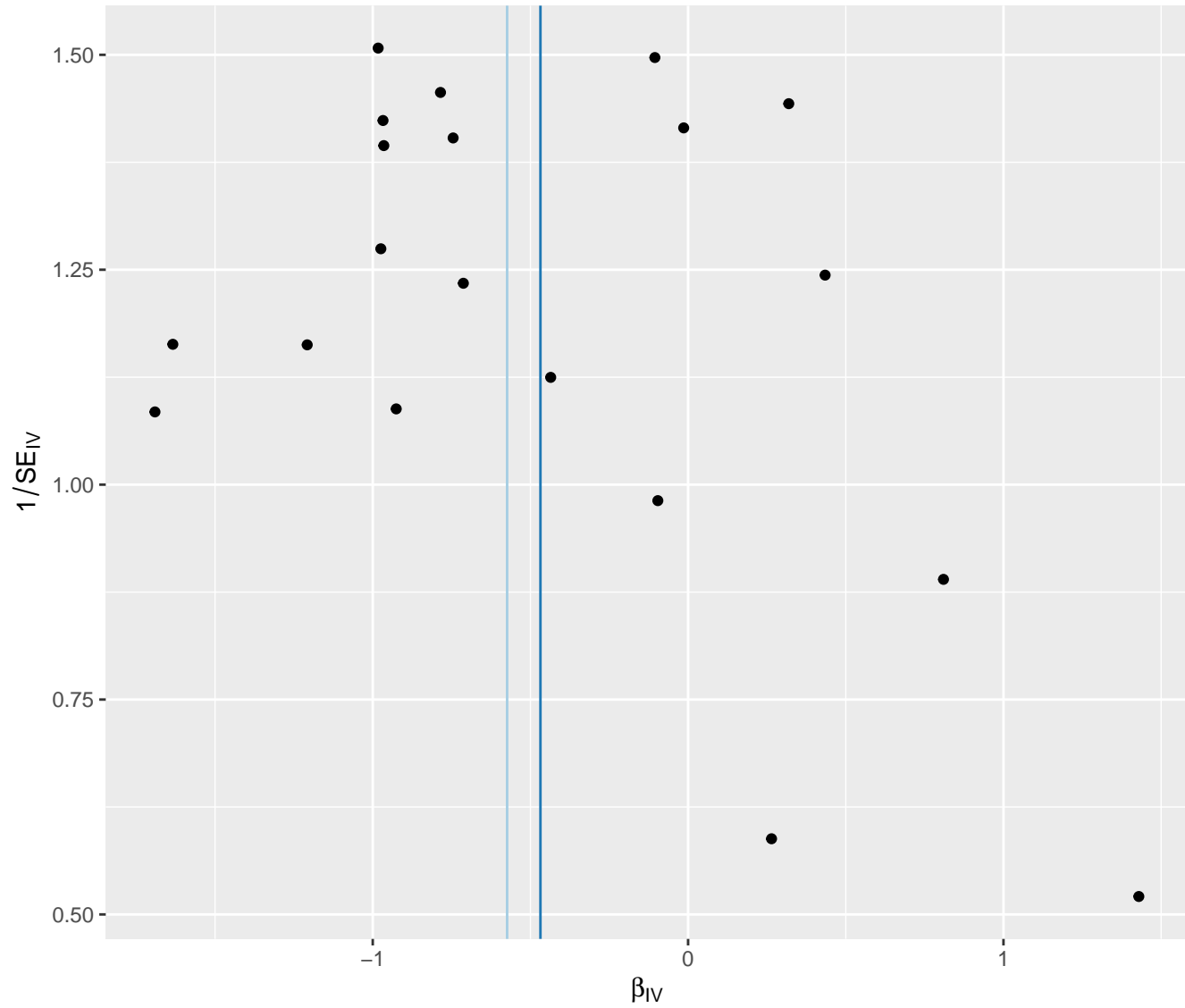

Supplement: Supplementary file 2 [file Presentation_1.zip › Supplementary Figure/Supplementary Figure35.pdf]

# MR Method

- Inverse variance weighted
- MR Egger

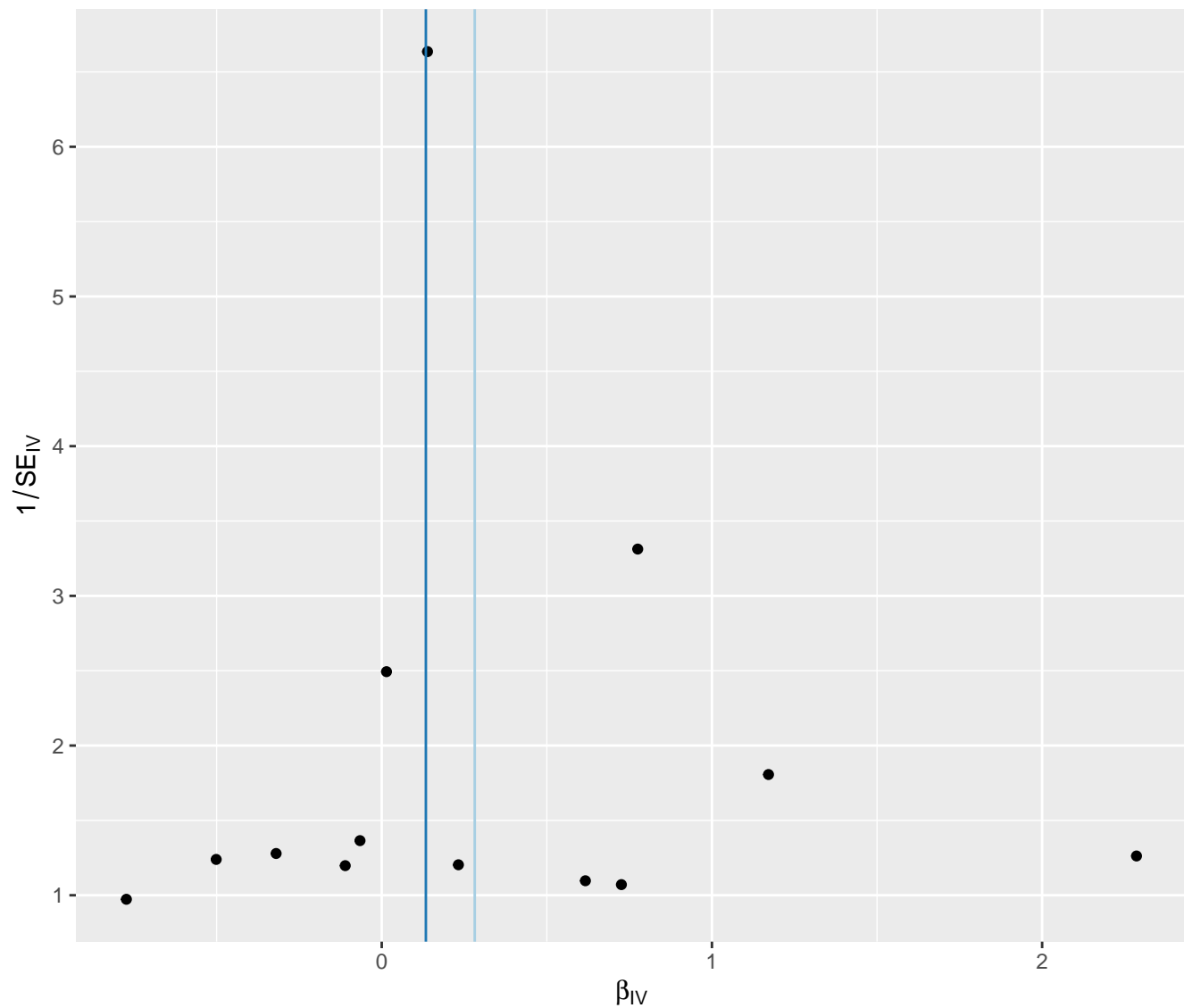

Supplement: Supplementary file 2 [file Presentation_1.zip › Supplementary Figure/Supplementary Figure36.pdf]

# MR Test

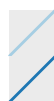

Inverse variance weighted

MR Egger

Weighted median

Weighted mode

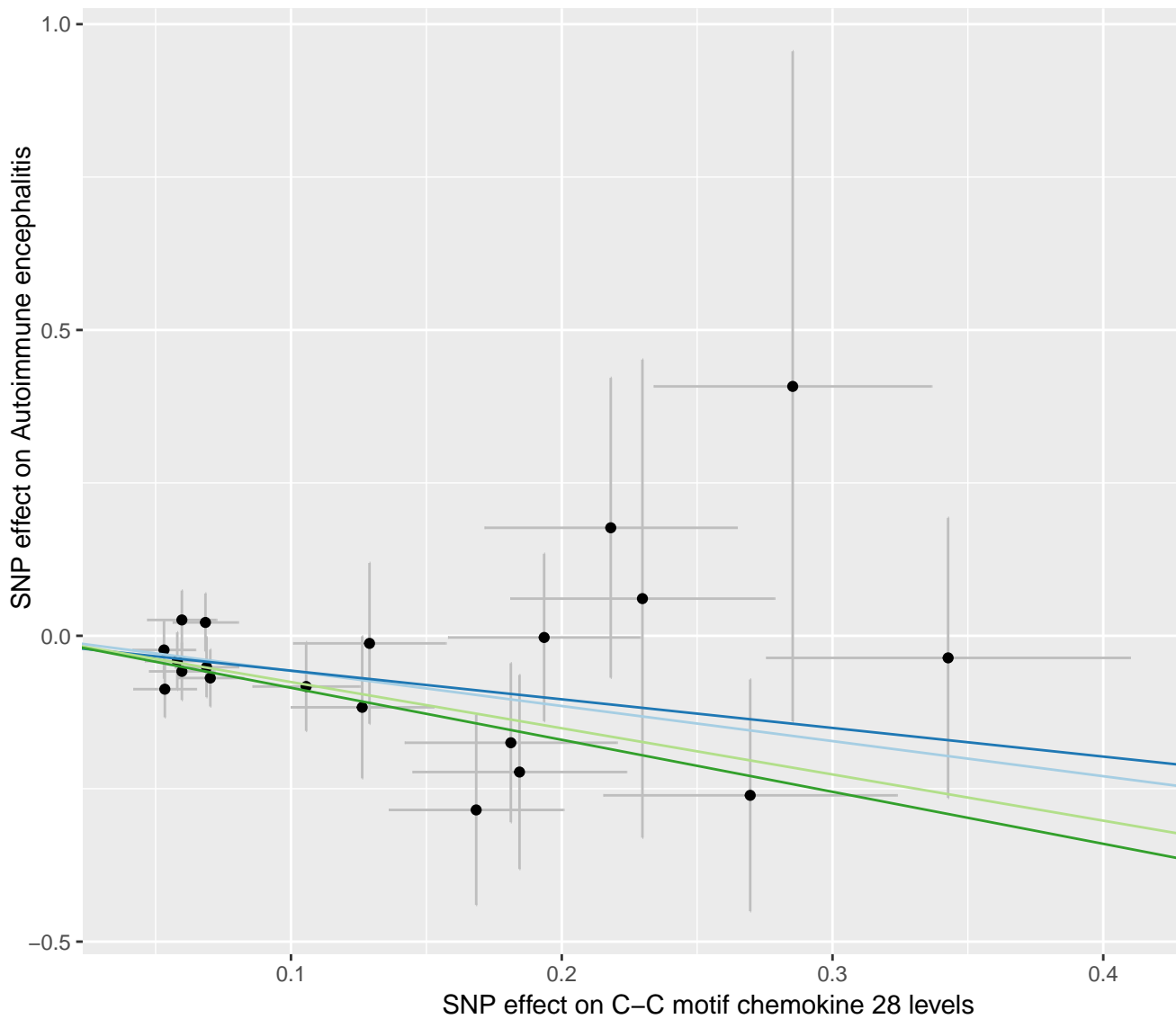

Supplement: Supplementary file 2 [file Presentation_1.zip › Supplementary Figure/Supplementary Figure37.pdf]

# MR Test

- Inverse variance weighted
- MR Egger
- Weighted median
- Weighted mode

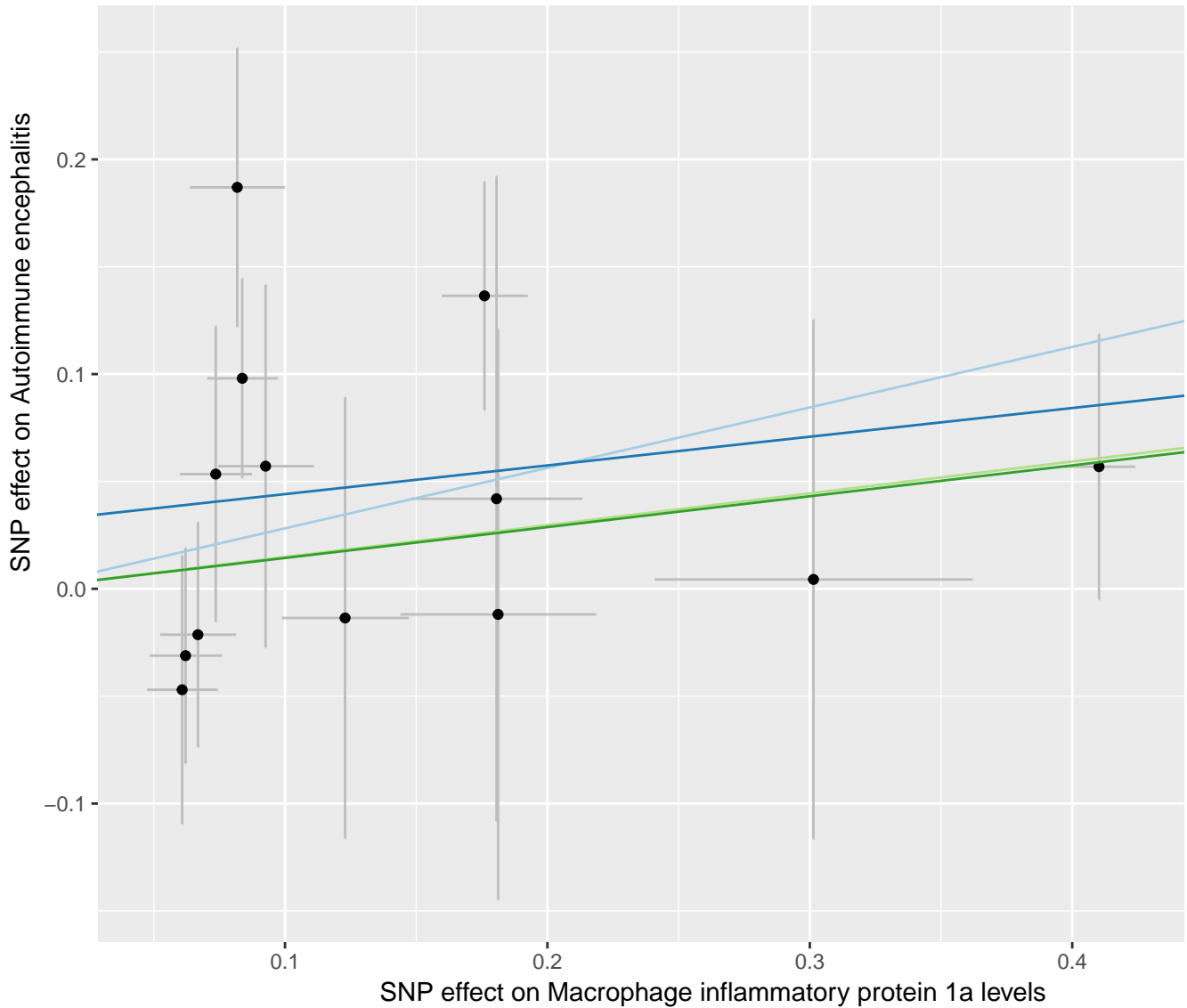

Supplement: Supplementary file 2 [file Presentation_1.zip › Supplementary Figure/Supplementary Figure38.pdf]

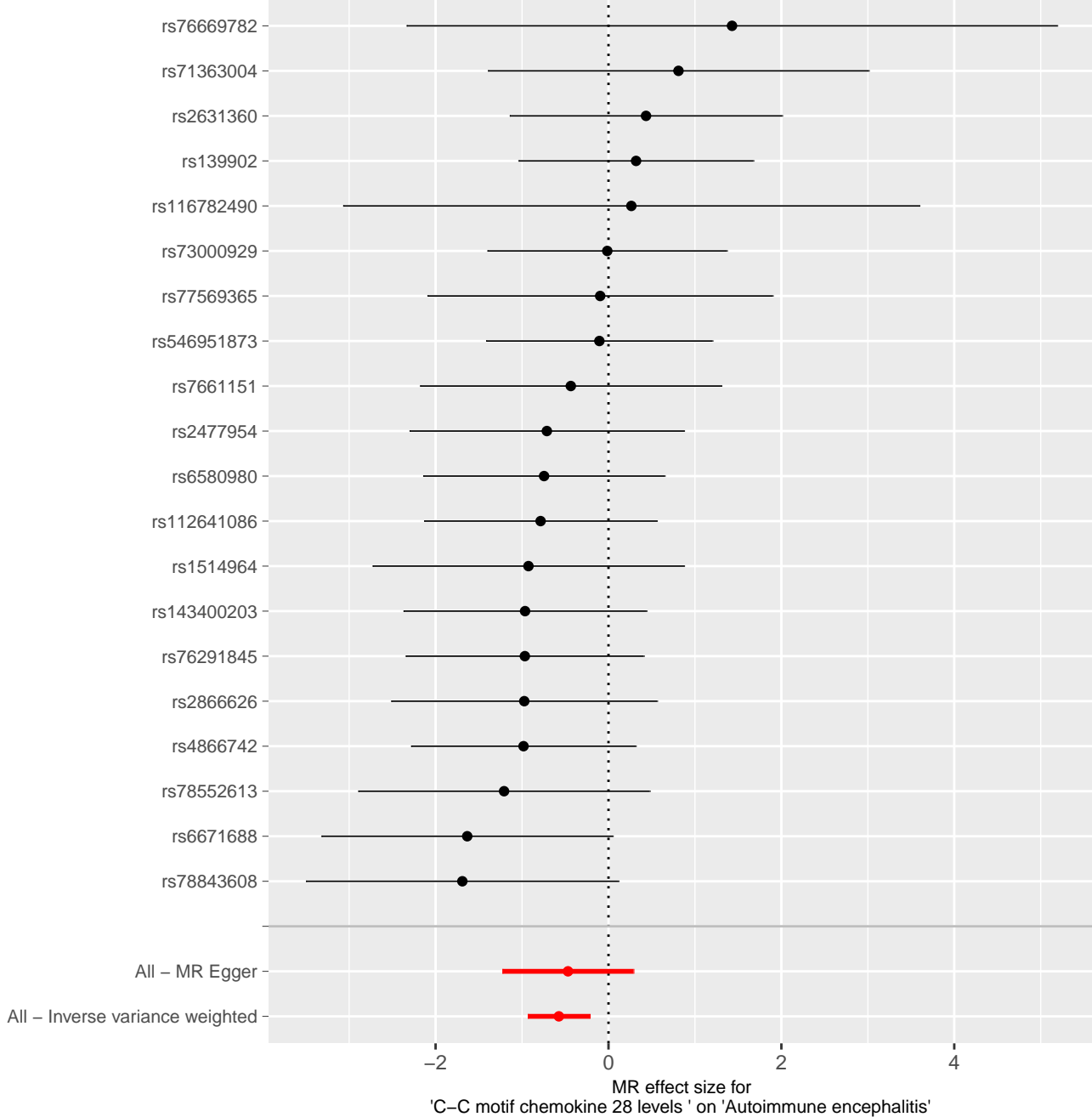

Supplement: Supplementary file 2 [file Presentation_1.zip › Supplementary Figure/Supplementary Figure39.pdf]

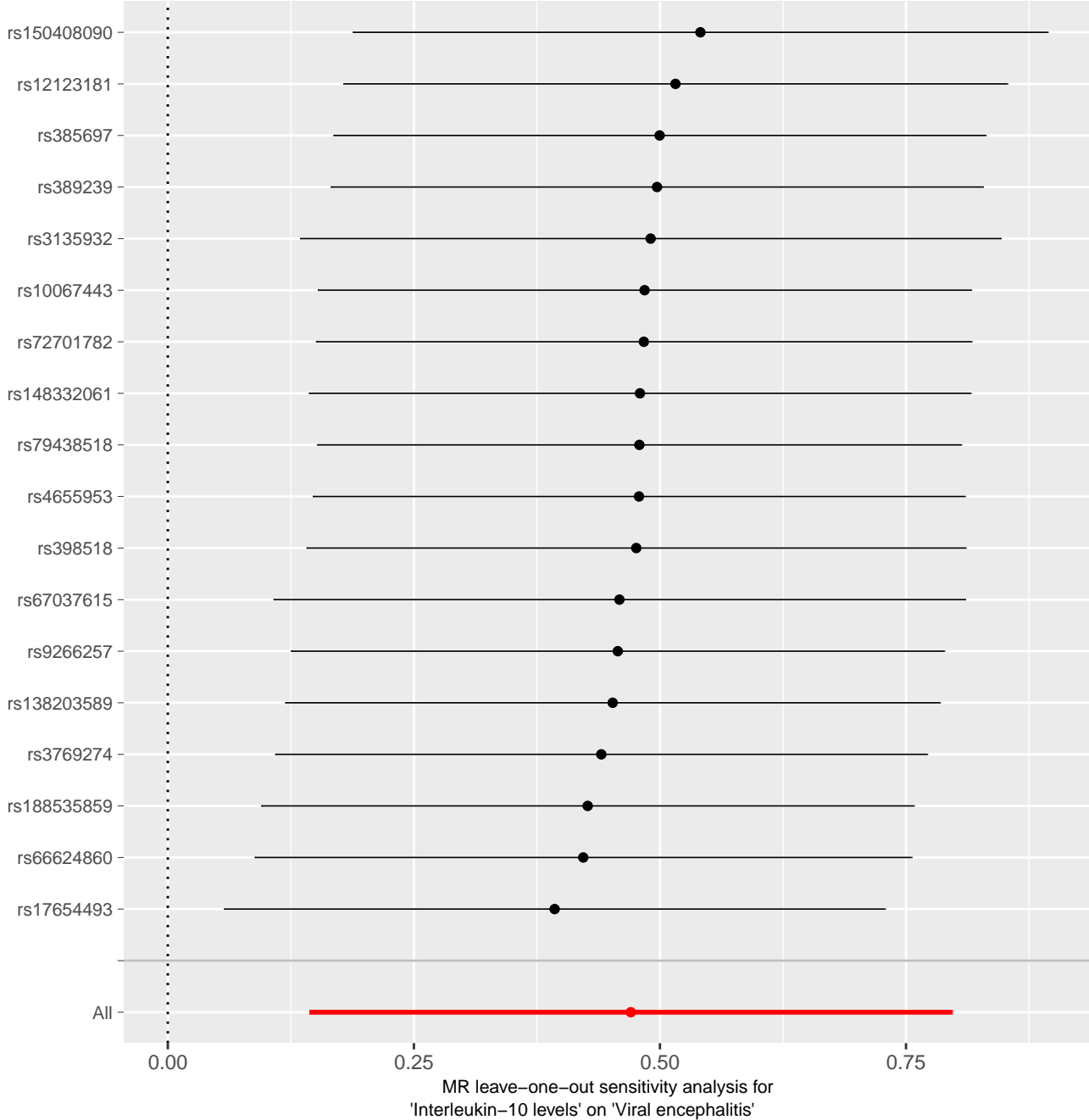

Supplement: Supplementary file 2 [file Presentation_1.zip › Supplementary Figure/Supplementary Figure4.pdf]

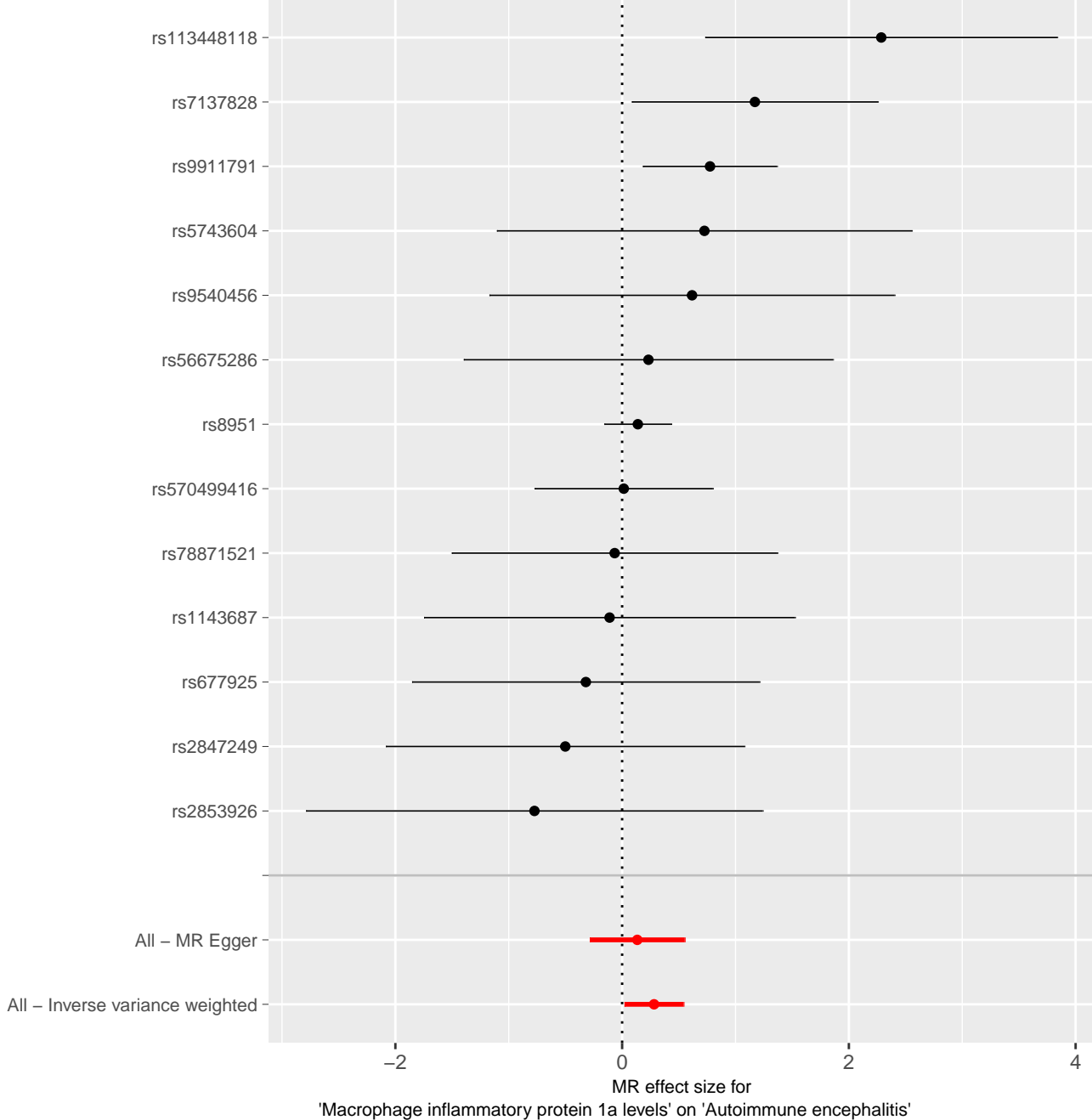

Supplement: Supplementary file 2 [file Presentation_1.zip › Supplementary Figure/Supplementary Figure40.pdf]

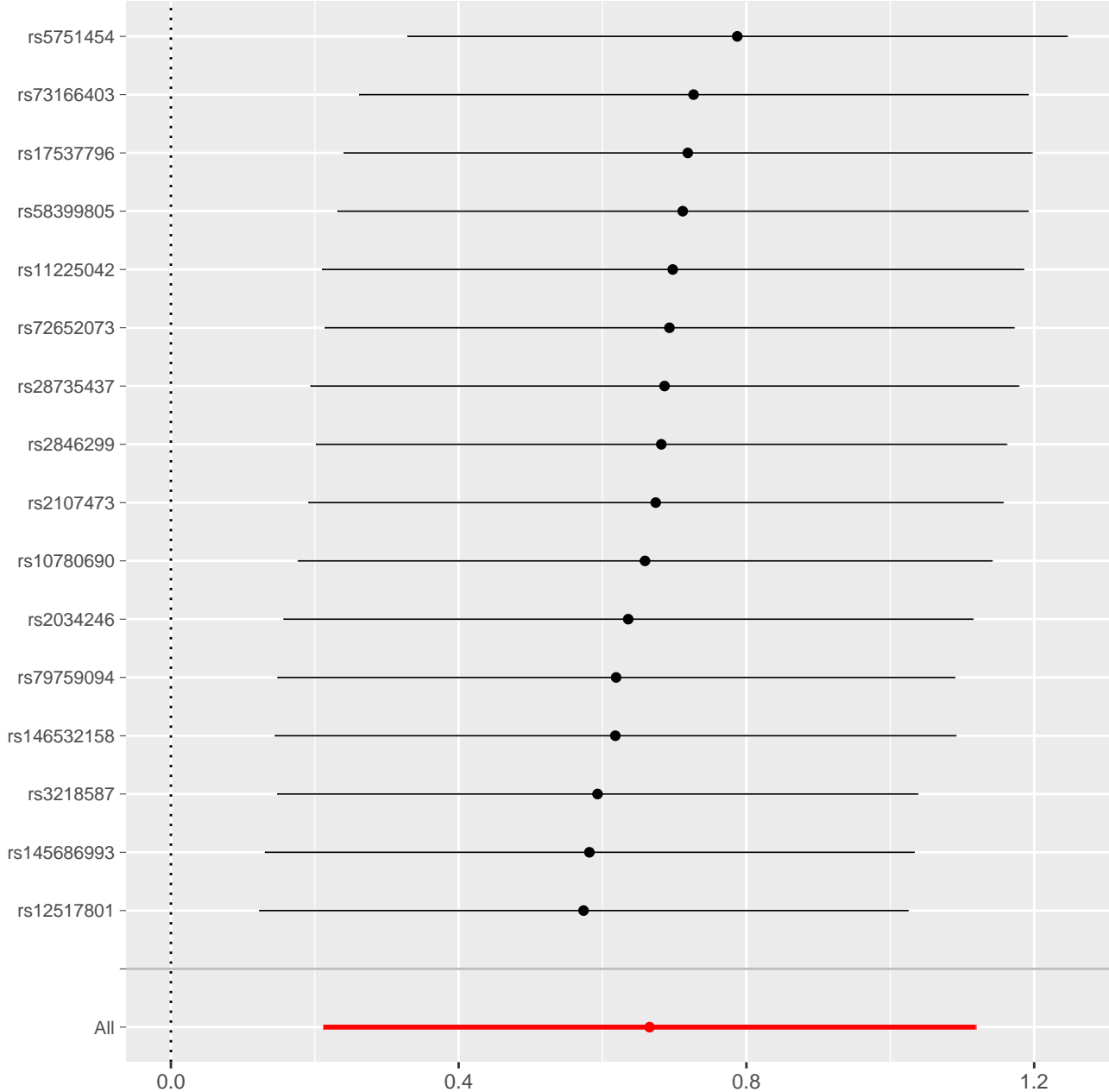

Supplement: Supplementary file 2 [file Presentation_1.zip › Supplementary Figure/Supplementary Figure5.pdf]

# MR Method

- Inverse variance weighted
- MR Egger

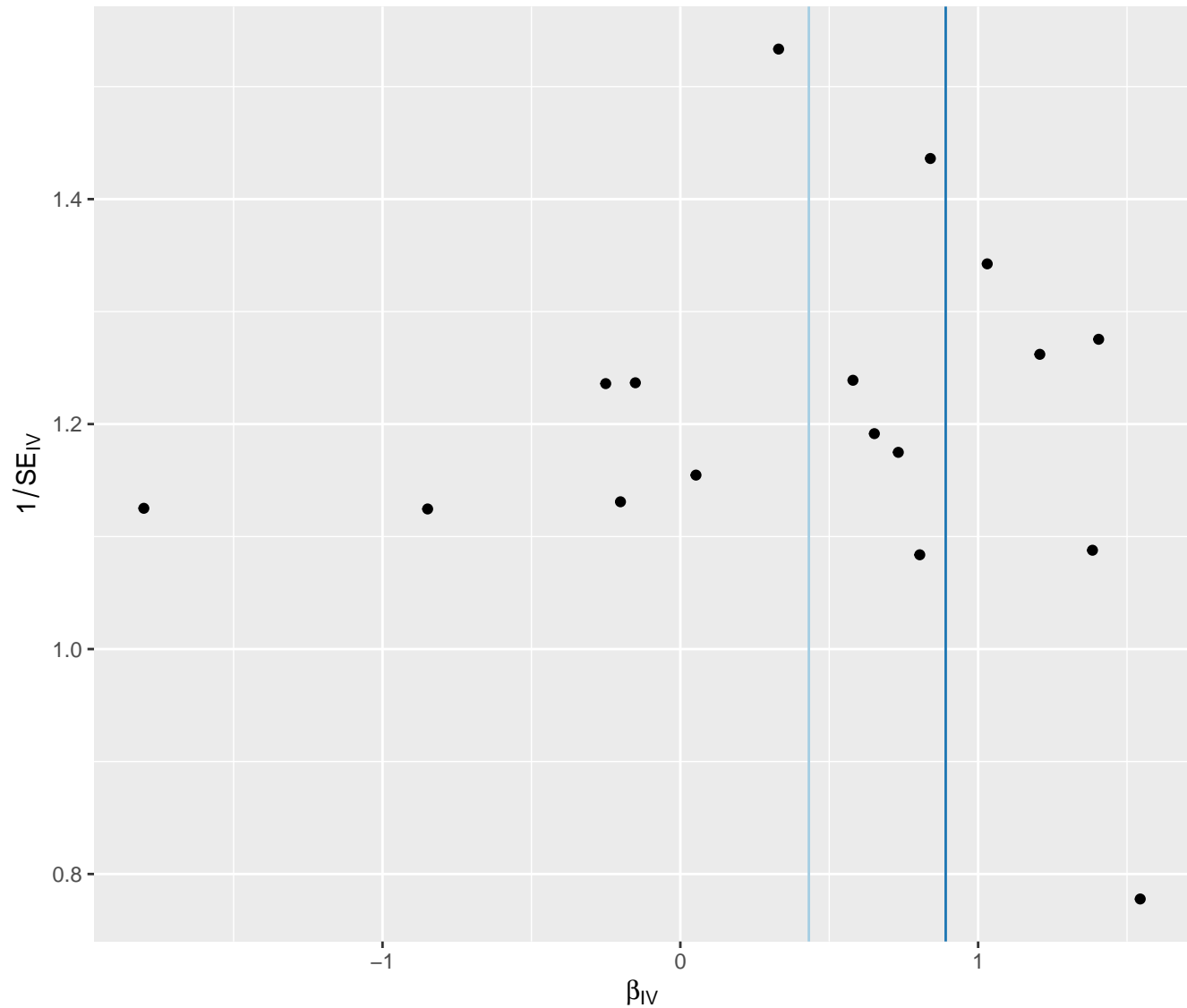

Supplement: Supplementary file 2 [file Presentation_1.zip › Supplementary Figure/Supplementary Figure6.pdf]

# MR Method

- Inverse variance weighted
- MR Egger

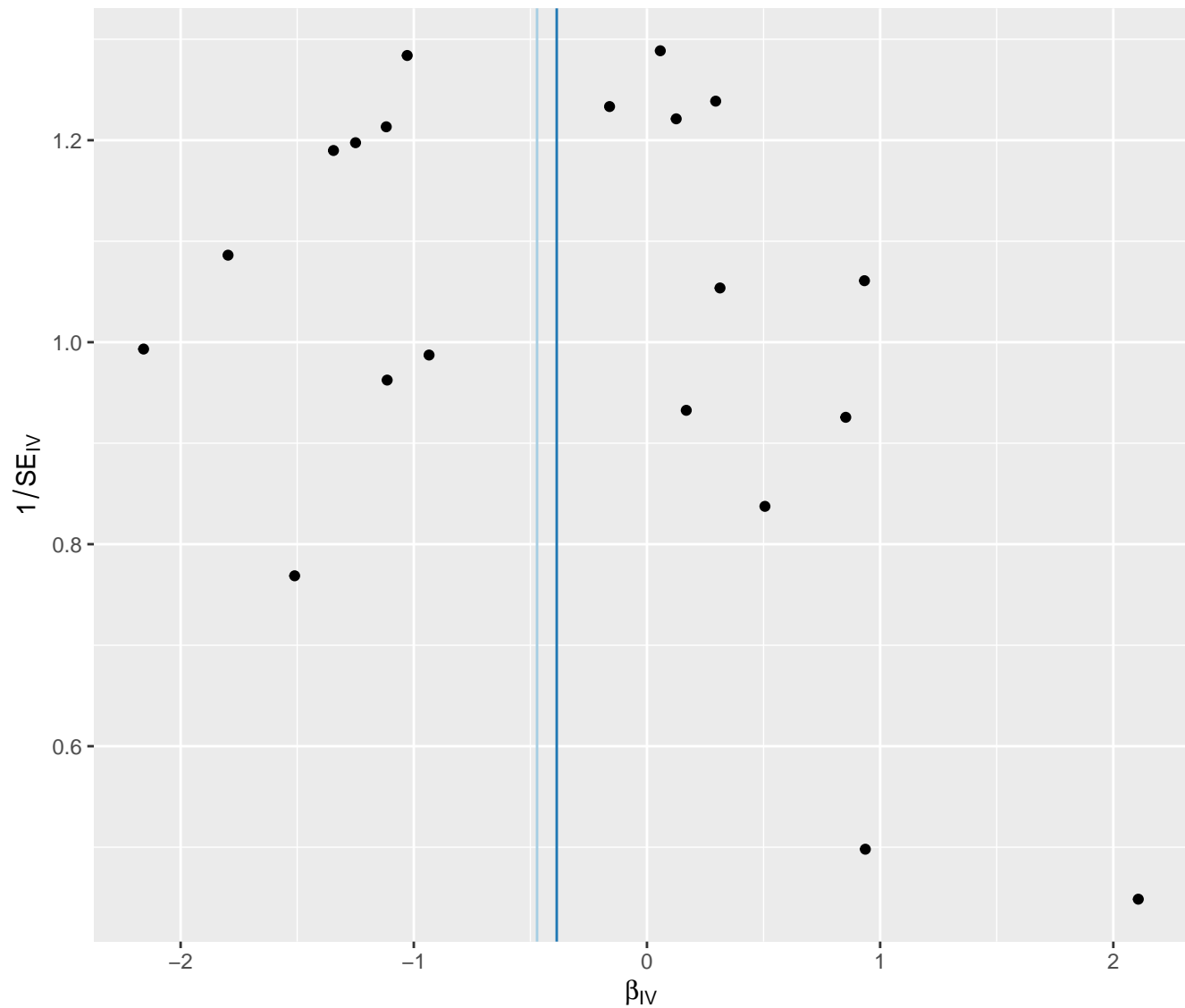

Supplement: Supplementary file 2 [file Presentation_1.zip › Supplementary Figure/Supplementary Figure7.pdf]

# MR Method

- Inverse variance weighted
- MR Egger

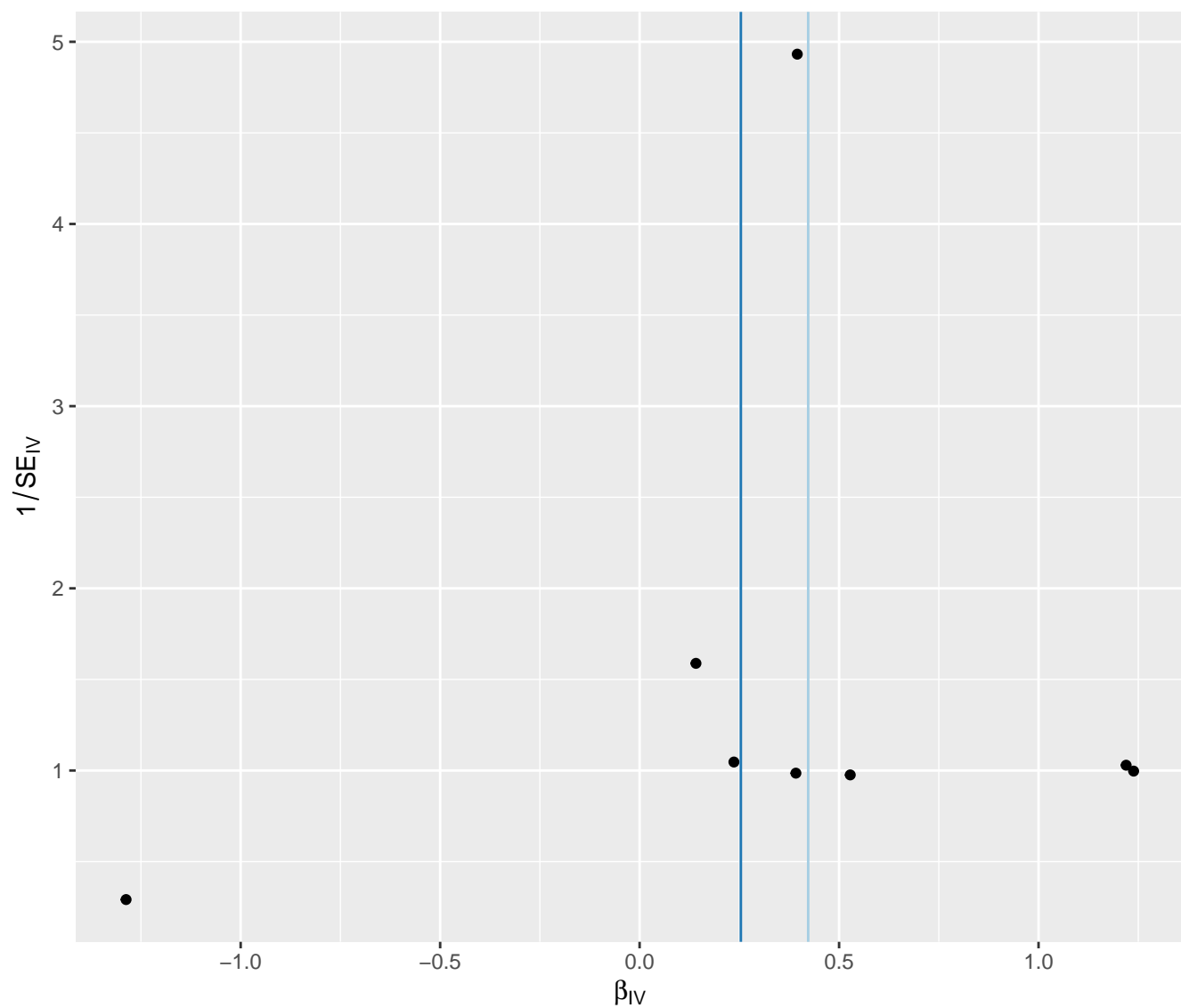

Supplement: Supplementary file 2 [file Presentation_1.zip › Supplementary Figure/Supplementary Figure8.pdf]

# MR Method

- Inverse variance weighted
- MR Egger

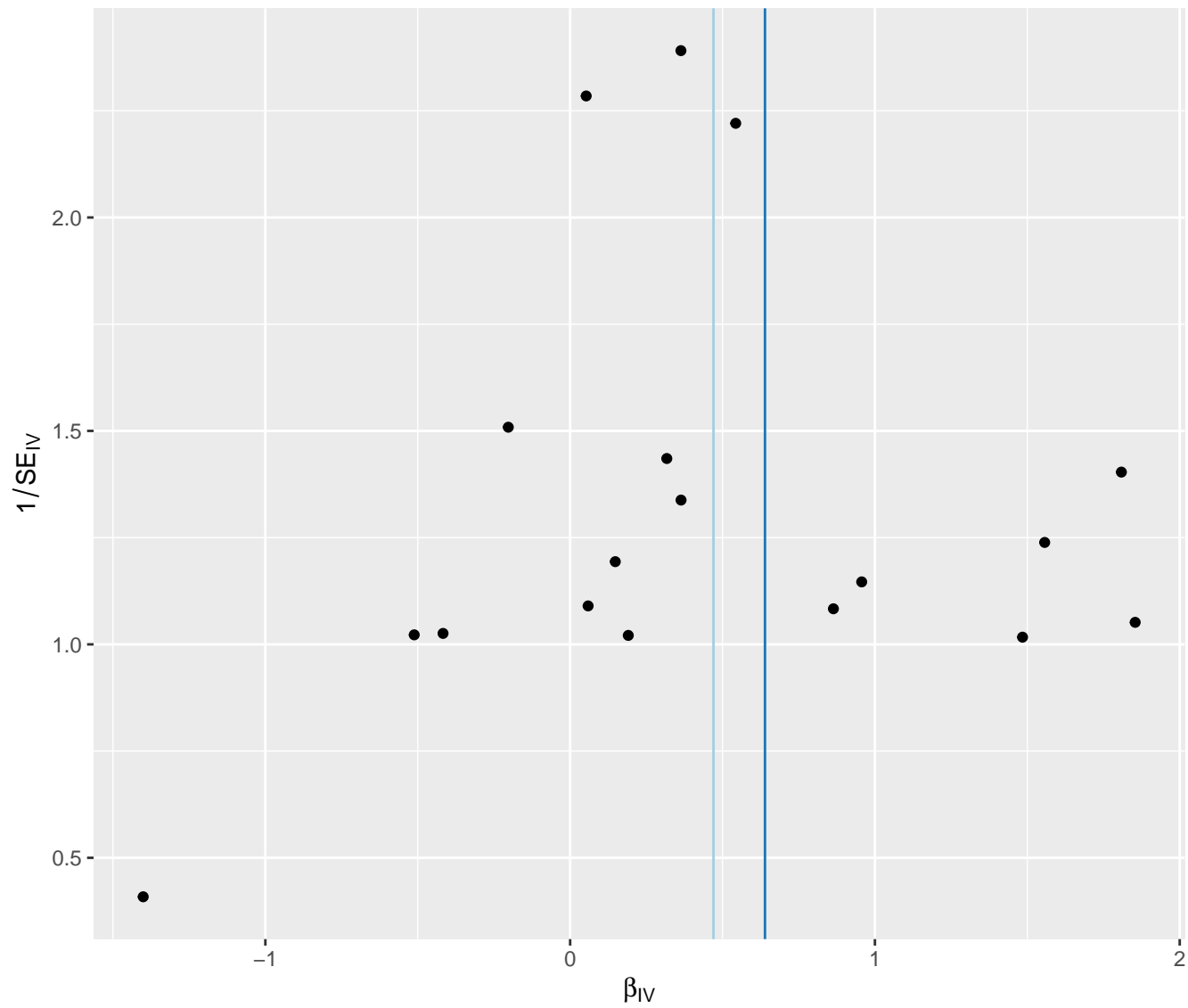

Supplement: Supplementary file 2 [file Presentation_1.zip › Supplementary Figure/Supplementary Figure9.pdf]
